# Development and validation of a TLS-associated signature for prognosis prediction in breast cancer: new insights into QPRT

**DOI:** 10.3389/fimmu.2026.1834127

**Published:** 2026-05-07

**Authors:** Qiao Li, Xin Yang, Lan Wei, Xing Wang, Xiaosong Wang, Junxia Chen, Liuyang Zhao, Siyang Wen

**Affiliations:** 1 Department of Laboratory Medicine, The Second Affiliated Hospital of Chongqing Medical University, Chongqing, China; 2 Department of Infectious Diseases, Key Laboratory of Molecular Biology for Infectious Diseases (Ministry of Education), Institute for Viral Hepatitis, The Second Affiliated Hospital, Chongqing Medical University, Chongqing, China; 3 Department of Cell Biology and Genetics, Chongqing Medical University, Chongqing, China; 4 Department of Laboratory Medicine, Chongqing Blood Center, Chongqing, China; 5 Department of Thyroid and Breast Surgery, The Second Affiliated Hospital of Chongqing Medical University, Chongqing Medical University, Chongqing, China; 6 Department of Gastroenterology, The First Affiliated Hospital of Chongqing Medical University, Chongqing, China; 7 Reproductive Medicine Center, The First Affiliated Hospital of Chongqing Medical University, Chongqing, China

**Keywords:** breast cancer, prognostic signature, QPRT, single cell RNA sequencing, tertiary lymphoid structures

## Abstract

**Background:**

Tertiary lymphoid structures (TLSs) are associated with superior prognosis in breast cancer (BC). TLSs serve as key niches of anti-tumor adaptive immune responses across various malignancies. However, the tumor-intrinsic factors that are associated with TLSs have been largely overlooked in BC.

**Methods:**

We integrated bulk and single-cell transcriptomic data to develop a TLS-related prognostic signature (TRPS) based on tumor-intrinsic TLS-related genes using machine learning algorithms. The predictive accuracy of the TRPS was validated through survival analysis, ROC curve evaluation, and the construction of a nomogram. The relationship between TRPS and the tumor microenvironment was assessed using TCGA-BC and in-house single-cell RNA-seq data. Furthermore, the relationship between TRPS and genomic alterations, drug sensitivity, and functional enrichment were explored. *In vitro* and *in vivo* functional assays were performed to investigate the role of QPRT, a key model gene, in the progression of BC. RNA sequencing, Western blotting, immunoprecipitation, immunofluorescence, immunohistochemistry, and flow cytometry were performed to elucidate the molecular mechanisms underlying the functions of QPRT.

**Results:**

The TRPS model exhibited robust prognostic performance, as validated across four independent cohorts. High-TRPS scores were associated with diminished infiltration of B cells and T cells. Moreover, the high-TRPS group displayed an elevated level of tumor mutation burden and enrichment of tumor-promoting pathways. QPRT facilitated BC growth and metastasis. Mechanistically, QPRT-mediated NAD^+^ accumulation promoted the deacetylation of β-catenin in a SIRT1-dependent manner, thereby activating Wnt/β-catenin signaling and upregulating PD-L1 expression. Notably, knockdown of QPRT sensitized BC to anti-PD-1 immunotherapy.

**Conclusions:**

This study establishes a novel TLS-related prognostic model, and highlights the potential of QPRT as a promising therapeutic target in BC.

## Introduction

Breast cancer (BC) remains the most prevalent malignancy and the second leading cause of cancer-related mortality among women (1). Immunotherapy, immune checkpoint inhibitors (ICIs), has revolutionized the treatment paradigms for several solid tumors (2). However, an immunosuppressive environment often hinders immunotherapeutic efficacy in BC. Therefore, it is crucial to comprehensively decipher the immune contexture of BC in order to enhance the clinical benefit of immunotherapy.

Tertiary lymphoid structures (TLSs) are ectopic lymphoid aggregates that play an important role in bolstering antitumor immunity (3). Accumulating evidence suggests that the presence of mature TLSs harboring germinal centers (GCs) correlates with improved outcomes in various cancers, such as pancreatic, bladder, gastric, and endometrial cancer (4–7). A TLS-derived gene signature has been shown to predict clinical outcomes in melanoma patients treated with ICIs (8). Notably, the prognostic significance of TLSs is largely predicated on their maturation and functional states, which are primarily driven by B cells (9). Moreover, the study identified a 9-gene TLS signature that is capable of evaluating TLS status and predicting improved clinical outcomes. In renal cell carcinoma, plasma cells (PCs) differentiated from mature B cells within TLSs secrete high-affinity IgG antibodies and induce tumor apoptosis, thereby predicting superior ICB response rates and progression-free survival (10). Similarly, TLS-associated B cells augment tumor-specific immune responses and correlate with improved prognosis in BC (11). Additionally, fibroblasts have been shown to recruit B cells through the CCL19-CCR7 axis to promote TLS assembly, which prolongs the survival of patients with colorectal cancer (12). Besides, in nasopharyngeal carcinoma, molecular signatures representing the core cellular components of TLSs (such as T follicular helper cells and cancer-associated fibroblasts) were derived from single-cell transcriptomic data, proving predictive of prognosis and immunotherapy responsiveness (13). On the other hand, the activity of TLSs can be suppressed by malignant cells, which negates their clinical benefits. For example, high-grade serous ovarian cancer cells can induce the transformation of normal mesenchymal stem cells (MSCs) into cancer-associated MSCs, which subsequently impede the formation of TLSs by inhibiting B-cell adhesion and proliferation (14). Additionally, cholesterol-biosynthetic tumor cells can hijack the MIF-CXCR4 interaction to disrupt GC reactions within TLSs, conferring immunotherapeutic resistance in esophageal squamous cell carcinoma (15). Despite the clinical significance of TLSs, TLS-associated tumor-intrinsic characteristics in BC remain elusive, and prognostic models leveraging such signatures have yet to be established.

Quinolinic acid phosphoribosyltransferase (QPRT) serves as a rate-limiting enzyme for the *de novo* synthesis of nicotinamide adenine dinucleotide (NAD^+^) (16). Notably, dysregulated NAD^+^ metabolism is linked to the development of various diseases, such as metabolic syndromes, senescence, and neurodegenerative pathologies (17). For example, QPRT deficiency impairs the *de novo* synthesis of NAD+, thereby exacerbating susceptibility to acute kidney injury in humans (18). A recent study has demonstrated that QPRT-mediated *de novo* NAD+ biosynthesis inhibits apoptosis, coffering treatment resistance in glioma cells (19). However, the role of QPRT-mediated NAD^+^ metabolism in BC progression and immune modulation remains elusive.

In this study, we identified 55 tumor-intrinsic, TLS-associated prognostic genes in BC by integrating transcriptomic data from both single-cell and bulk cohorts. Based on these candidate genes, we employed machine learning methods to construct a novel TLS-related prognostic signature (TRPS) composed of three genes: CD24, QPRT, and SPINT1. The predictive performance of the TRPS was validated across 4 independent BC datasets. This model was further evaluated in the context of the TME, genomic alterations, and drug sensitivity. Moreover, the expression and immune infiltration patterns associated with the TRPS were corroborated at single-cell resolution. Through comprehensive *in vitro* and *in vivo* experiments, we investigated the role and underlying mechanisms of QPRT in facilitating BC progression. Our study provides a robust framework for improving risk stratification and underscores the potential of QPRT as a therapeutic strategy for BC.

## Materials and methods

### Data acquisition

Clinical, transcriptomic, and genomic profiles of BC were integrated from The Cancer Genome Atlas (TCGA) database, GEO (GSE246613, GSE20685, GSE42568, and GSE96058), and METABRIC (via cBioPortal) (20, 21). The TCGA-BC somatic mutation data (MAF format) were sourced from UCSC Xena (22), and processed via the “maftools” package. Samples lacking complete overall survival (OS) data or genes expressed in less than half of the samples were excluded.

### Development and validation of the TRPS

First, differentially expressed genes (DEGs) of malignant cells between TLS-positive and TLS-negative samples were identified (|log_2_FC| > 0.25, min.pct > 0.1) using the “Seurat” package. In parallel, DEGs between tumor and normal tissues were identified (|log_2_FC| > 1, adjusted *P* value < 0.05) via the “edgeR” package based on the TCGA-BC cohort. 448 TLS-related genes were obtained by intersecting these DEGs.

Subsequently, univariate Cox regression was employed to select genes significantly associated with overall survival (OS). The Least Absolute Shrinkage and Selection Operator regression analysis (LASSO) regression analysis via “glmnet” package (23) and random survival forests (RSF) analysis using “randomForestSRC” package were further performed for refinement. Finally, stepwise Cox proportional hazards regression analysis was conducted to generate the TLS-related prognostic signature (TRPS), the calculation of which is as follows:


Risk score = (0.129 × QPRT) + (0.283 × SPINT1) + (0.161 × CD24)

The time-dependent receiver operating characteristic (ROC) curves and Kaplan-Meier survival curves were employed for model evaluation. To enhance the clinical utility of the TRPS model, a prognostic nomogram was developed using the “rms” package, and decision Curve analysis (DCA) was conducted to determine its clinical net benefit via the “ggDCA” package.

### Immune landscape and drug sensitivity analysis

The overall infiltration status was first analyzed using the “ESTIMATE” package. Additionally, five algorithms (TIMER, CIBERSORT, quanTIseq, MCPcounter, and EPIC) were performed to comprehensively evaluate the infiltration of various cell types into the TME (24). Drug sensitivity was predicted based on the IC50 values of individual antitumor agents computed using the “oncoPredict” package (25).

### Functional enrichment analysis

To systematically evaluate biological pathway variations between the risk subgroups, two complementary approaches were employed. Gene Set Variation Analysis (GSVA) was performed using the “GSVA” package to calculate sample-wise enrichment scores for the Hallmark gene sets (v2025.1) from the Molecular Signatures Database (MSigDB). Subsequently, differential pathway activity was determined using the “limma” package, where pathways with an absolute *t*-value > 2 and a *P*-value < 0.05 were identified as significantly enriched.

Additionally, Gene Set Enrichment Analysis (GSEA) was conducted using the “clusterProfiler” package (26). Pathways with a *P*-value < 0.05 were considered significantly enriched. Finally, the results were visualized using the “enrichplot” package.

### Single-cell RNA sequencing and analysis

Fresh human BC tissues were digested in DMEM containing Collagenase P (2 mg/mL; Roche), Dispase II (1 mg/mL; Yeasen) and DNase I (0.2 mg/mL; Yeasen) at 37°C for 30 minutes, and filtered using 70 μm cell strainers (Biofil, China). Red blood cells were subsequently lysed on ice using ACK lysis buffer (Gibco, USA) for 3 minutes. After cell viability and concentration determination using a Rigel S2 automated cell counter (Countstar), qualified single-cell suspensions were loaded onto microfluidic chips for droplet-based partitioning, followed by library construction. Finally, libraries were pooled and sequenced on GeneMind SURFSeq 5000 (paired-end, 150 bp).

For quality control, cells with 300-7, 000 detected genes and fewer than 20% mitochondrial content, along with genes expressed in at least 5 cells, were retained for downstream analysis. After batch correction via Harmony (27), SNN clustering and t-SNE visualization were conducted based on the top 20 principal components (28). Cell types were annotated using canonical marker genes (29–31), and the “AUCell” algorithm was applied to score the TRPS at single-cell level (32). To assess the association between the TRPS and TLS status, the “AddModuleScore” function in Seurat was employed to compute TLS-related gene signatures that recapitulate the transcriptomic patterns associated with TLSs, including a 9-gene TLS signature (8), a 12-Chemokine TLS signature (33), a 34-gene HEV signature (34), and a GC signature (35) (**Supplementary Table 1**). Additionally, the cellular interactions were inferred using the “CellChat” package (36).

### Cell culture and lentiviral transduction

Breast cancer cell lines and normal mammary epithelial cell MCF-10A were obtained from the American Type Culture Collection (ATCC). Specifically, MDA-MB-231, MCF-7, and E0771 cells were maintained in DMEM (Gibco, USA). BT-549 and T-47D cells were cultured in RPMI-1640 medium (Gibco, USA). MDA-MB-453 cells were grown in Leibovitz’s L-15 medium (Gibco), and SK-BR-3 cells were maintained in McCoy’s 5A medium (Gibco, USA). All aforementioned media were supplemented with 10% fetal bovine serum (FBS; Gibco) and 1% penicillin-streptomycin (P/S; Biosharp, China). MCF-10A cells were cultured in specialized DMEM/F12 medium (Procell, China) supplemented with 5% horse serum, 0.5 μg/mL hydrocortisone, 10 μg/mL insulin, 20 ng/mL epidermal growth factor (EGF), 1% non-essential amino acids (NEAA), and 1% P/S. All cells were maintained at 37°C in a humidified atmosphere with 5% CO_2_.

The lentiviral vectors for knockdown and overexpression of QPRT were synthesized by Genechem. To construct stable knockdown cell lines, BC cells were infected with lentiviruses expressing shRNAs targeting human QPRT or murine Qprt, while a non-targeting shRNA served as a negative control. For QPRT overexpression, BC cells were transduced with lentiviruses carrying the human QPRT coding sequence or an empty vector. The infected cells were subsequently subjected to selection by 2 μg/mL puromycin (Beyotime, China) for two weeks. Finally, the efficiency of QPRT knockdown and overexpression was validated via Western blotting. The detailed oligo sequences used in this study are listed in **Supplementary Table 2**.

### Western blotting assay

Total proteins were extracted using RIPA buffer and quantified via the BCA assay. The proteins were resolved by SDS-PAGE, transferred onto PVDF membranes, and blocked with 5% nonfat milk. Membranes were incubated with primary antibodies and corresponding HRP-conjugated secondary antibodies against QPRT (Proteintech, China), Non-phospho (Active) β-Catenin (CST, USA), β-Catenin (CST), c-Myc (Proteintech), Cyclin D1 (Proteintech), PD-L1 (Proteintech), and β-actin (Proteintech) at 4°C overnight, followed by incubation with corresponding secondary antibodies at room temperature (RT) for 1 hour. Finally, the protein bands were visualized using Enhanced ECL (Yeasen, China) on ChemiDoc XRS^+^ system (Bio-Rad, USA). Antibody details are provided in **Supplementary Table 3**.

### Proliferation detection

Proliferation was assessed via colony formation assay and EdU incorporation assay. For colony formation, 500 transfected cells were seeded per well into 6-well plates and allowed to grow for two weeks. After being fixed with 4% paraformaldehyde (PFA), colonies were stained with 0.5% crystal violet for visualization and quantified with ImageJ software. EdU-based cell proliferation assay was performed according to the manufacturer’s instructions (Cellorlab, China), and cells were imaged under a fluorescence microscope (Leica, Germany).

### Transwell invasion assay

The 8-μm pore upper chambers (Corning, USA) were pre-coated with Matrigel (Corning, USA) at 37°C for 30 minutes. Cells resuspended in 200 μL serum-free DMEM were seeded into the upper chamber at a density of 20, 000 cells/well, with the lower chamber filled with 500 μL DMEM containing 10% FBS. The cells were then allowed to invade the chamber for 24 hours. Subsequently, invaded cells were fixed with 4% PFA (Solarbio, China) and stained with 0.1% crystal violet. Images were captured under a microscope (Nikon, Japan) and the invaded cells were counted in three random fields.

### Flow cytometry analysis

For cell apoptosis detection, 2 × 10^5^ BC cells were harvested, incubated with PI/Annexin V-FITC staining buffer, and then detected on a CytoFLEX flow cytometer (Beckman Coulter, USA).

For detection of CD8^+^ T cells, fresh tumor samples were digested with 0.1 mg/mL collagenase IV and 0.1 mg/mL DNase I, blocked with purified anti-Mouse CD16/32 antibody, stained with CD3, CD8a and CD45 antibodies, and analyzed on a CytoFLEX flow cytometer.

For detection of cell surface PD-L1 in BC cells, cells were harvested and washed twice with pre-cooled PBS and adjusted to a density of 1 × 10^6^ cells/mL. Then, an aliquot of 100 μL cell suspension was incubated with FITC-conjugated anti-human PD-L1 antibody (Elabscience, China) for 30 min on ice in the dark, following which the cells were washed. The fluorescence signals were acquired on a flow cytometer, and the data were analyzed using FlowJo software to determine the mean fluorescence intensity (MFI).

### Bulk RNA sequencing assay

Total cellular RNA was isolated using TRIzol reagent (Invitrogen, USA) and reverse-transcribed into cDNA, which was subsequently end-repaired and ligated to barcoded sequencing adapters to construct sequencing libraries. The library quality was evaluated using an Agilent 2100 Bioanalyzer, and qualified libraries were then pooled and subjected to high-throughput sequencing.

### RNA extraction and qRT-PCR

Total RNA was isolated using the TRIzol reagent, followed by cDNA synthesis via the HiScript II Q RT SuperMix (Vazyme, China) on a T100 Thermal Cycler (Bio-Rad, USA). Quantitative PCR was subsequently performed utilizing TB Green Premix Ex Taq (Takara, China) according to the manufacture’s protocols. The 2^-ΔΔct^ approach was employed to quantify the relative abundance of mRNA, with β-actin serving as the internal normalization control. All primer sequences are documented in **Supplementary Table 2**.

### Immunoprecipitation and acetylation detection

The Pierce Classic Magnetic IP/Co-IP Kit (Thermo Fisher Scientific, USA) was used to conduct immunoprecipitation (IP) following the manufacturer’s instructions. Briefly, 1 × 10^7^ BC cells were harvested and lysed in the IP lysis/wash buffer on ice for 20 minutes. Following centrifugation, a portion of the supernatant was reserved as the input control. The remaining lysates were incubated with primary antibody against β-catenin or IgG at 4 °C overnight. The immunocomplexes were then captured by incubation with Protein A/G Magnetic Beads for 4 hours at 4 °C. The beads were subsequently collected using a magnetic stand and washed three times with pre-chilled lysis buffer. Then, the precipitated proteins were eluted with IP lysis/wash buffer for 5 minutes. Finally, the enriched β-catenin proteins were subjected to western blotting to detect their acetylation status.

### Measurement of intracellular NAD^+^ levels

Intracellular NAD^+^ concentrations were determined utilizing a NAD^+^/NADH colorimetric assay kit (Elabscience, China) following the manufacturer’s protocol. Briefly, 1.5 × 10^6^ BC cells were harvested, and the resulting cell lysates were partitioned for separate analyses. To quantify total NAD levels, 20 μL of the supernatant was incubated with the reaction working solution at 37 °C for 30 min. For specific NADH detection, the NAD^+^ fraction was first eliminated by pre-heating the samples at 60 °C for 30 minutes. Absorbance was recorded at 450 nm using a microplate reader. NAD^+^ content was derived by subtracting the NADH value from the total NAD pool. All results were normalized to the total protein concentration of each sample.

### Nuclear and cytoplasmic fractionation assay

Subcellular fractionation was performed using the PARIS™ Kit (Thermo Fisher Scientific, USA) following the manufacturer’s instructions. Briefly, cells were lysed in ice-cold cell fractionation buffer to selectively isolate the cytoplasmic fraction, followed by treatment of the remaining nuclear pellet with cell disruption buffer to extract nuclear proteins. The isolated protein fractions were subsequently subjected to Western Blotting analysis, with GAPDH and Histone H3 utilized as loading controls for the cytoplasmic and nuclear compartments, respectively.

### Immunofluorescence

BC cells were seeded on coverslips. When cultured to 50% confluence, the cells were fixed with 4% PFA for 15 minutes at RT, permeabilized with 0.1% saponin, and blocked using 4% bovine serum albumin (BSA) for 1 hour. Cells were incubated overnight at 4 °C with primary antibodies. After washing three times with PBS, the cells were incubated with fluorescence-conjugated secondary antibody for 2 hours at RT. Nuclei were counterstained with DAPI. Finally, the coverslips were mounted onto glass slides using an anti-fade mounting medium (Invitrogen) and visualized on a Leica confocal microscope.

### Histopathological analysis

Formalin-fixed and paraffin-embedded (FFPE) lung tissue sections were stained with hematoxylin and eosin (H&E) and assessed for metastatic nodules of breast cancer. For immunohistochemistry (IHC) staining, 4-μm-thick FFPE BC tissue sections were dewaxed, rehydrated, and retrieved for antigen. Subsequently, the tissues were covered with primary antibodies at 4 °C overnight, followed by incubation with secondary antibodies for 2 hours at RT. Finally, the sections were stained with diaminobenzidine and hematoxylin.

### Human specimens

The treatment-naïve clinical BC tissue samples were collected from patients receiving treatment at the Second Affiliated Hospital of Chongqing Medical University (CQMU), upon approval from the Ethics Committee of CQMU.

### Animal studies

For orthotopic tumor models, 1 × 10^6^ MDA-MB-231 or 5 × 10^5^ EO771 cells were inoculated into the mammary fat pads (MFP) of 6-week-old nude or C57BL/6 female mice, respectively. For metastasis, 1 × 10^6^ MDA-MB-231-Luc cells were injected via the tail vein, and the lung metastatic burden was assessed by bioluminescence imaging (BLI) (NightOWL LB 983, Berthold). For immunotherapy, E0771-bearing mice received anti-PD-1 or an IgG2a control (100 μg, intraperitoneally [I.P.], BioXcell) every 3 days starting from 11 days post-inoculation. Tumor volume was calculated as 0.5 × length × width^2^. All procedures were approved by the Ethics Committee of CQMU.

### Statistical analysis

R software (v4.4.3) and GraphPad Prism (v10.1.1) were utilized to perform statistical analysis. Intergroup differences between two groups were assessed using Student’s t-test or Wilcoxon rank-sum test as appropriate. Differences among multiple groups were analyzed by ANOVA. For Kaplan-Meier survival analysis, statistical significance was determined by the log-rank test. A two-sided *P* < 0.05 was considered statistically significant. All experimental assays were performed in triplicate.

## Results

### Construction of the TLS-related prognostic signature in breast cancer

**Figure 1:**
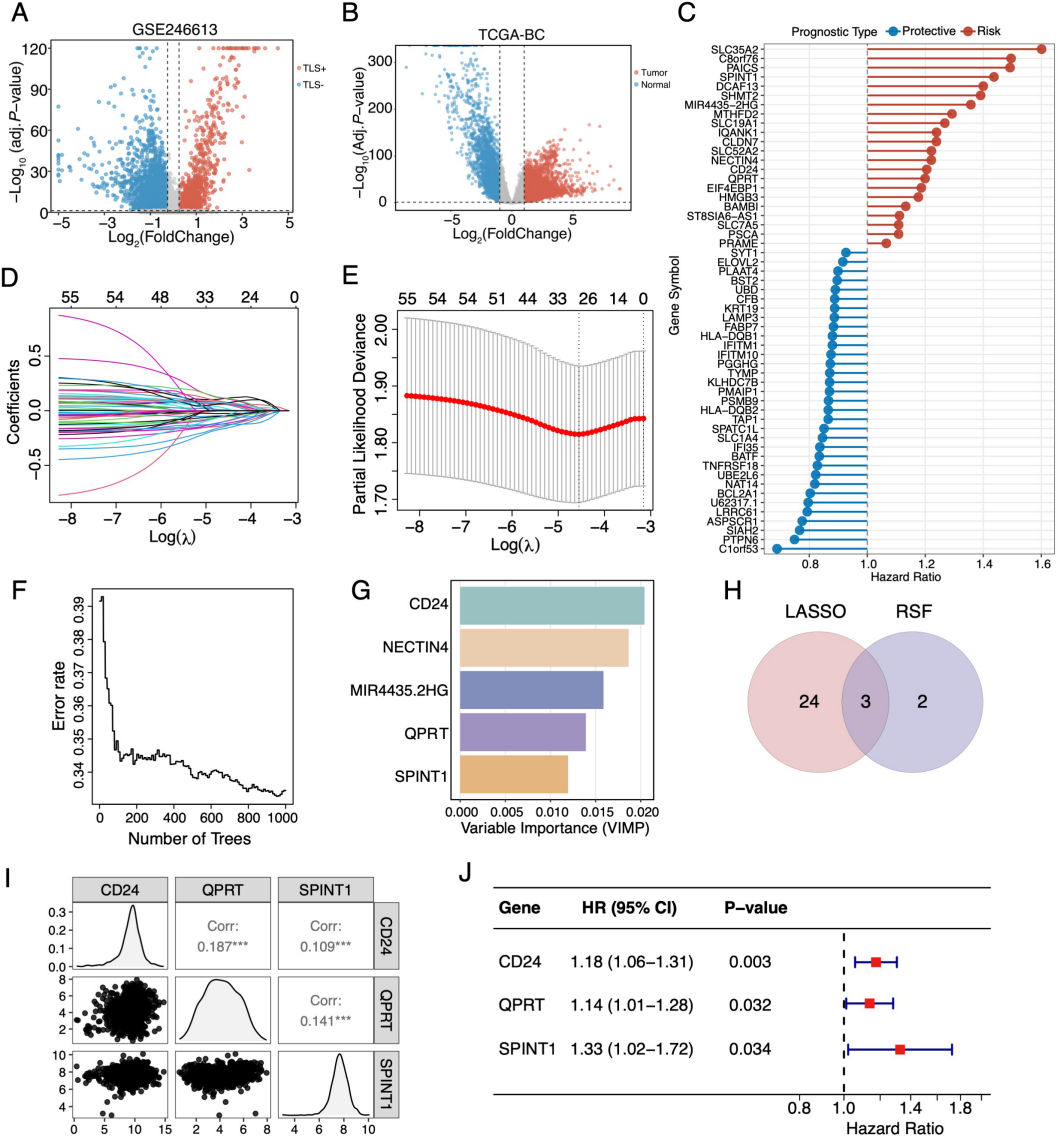
Development of a TLS-related prognostic signature in BC. **(A)** Volcano plot of differentially expressed genes (DEGs) of malignant cells identified between TLS-positive and TLS-negative BC samples. **(B)** Volcano plot illustrating DEGs between tumor and adjacent normal tissues in the TCGA-BC cohort. **(C)** Lollipop plot illustrating TLS-associated genes significantly associated with overall survival (OS). **(D)** LASSO coefficient profiles of the candidate genes. **(E)** Selection of the optimal tuning parameter (λ) via ten-fold cross-validation. **(F)** Error rate of the Random Survival Forests (RSF) algorithm. **(G)** Ranking of the top candidate genes identified by the RSF algorithm. **(H)** Venn diagram intersecting LASSO- and RSF-selected genes. **(I)** Pairwise scatterplot matrix depicting the correlation pattern and expression distributions of the model genes. ^***^p < 0.001. **(J)** Forest plot visualizing the hazard ratio (HR) of the three model genes comprising the signature.

To systematically identify cancer cell-specific genes associated with TLSs in BC, we first analyzed the scRNA-seq dataset GSE246613 (**Supplementary Figures 1A, B**) (37). Compared to TLS-negative samples, TLS-positive samples exhibited a distinct immune-enriched TME, characterized by increased proportions of T and B cells, decreased malignant cells, and markedly elevated TLS signature scores [as defined by Coppola et al. (33)] (**Supplementary Figures 1C, D**). Next, we identified DEGs of malignant cells between TLS-positive and TLS-negative groups (**Figure 1A**), and DEGs upregulated in tumor tissues from the TCGA-BC cohort (**Figure 1B**). The overlapping genes were then subjected to univariate Cox regression analysis, obtaining a total of 55 candidate genes with prognostic value (**Figure 1C**). Subsequently, LASSO-Cox regression (**Figures 1D, E**) and Random Survival Forests (RSF) analysis (**Figures 1F, G**) were employed for further refinement, yielding three overlapping genes (**Figure 1H**). Ultimately, an optimized TLS-related prognostic model (TRPS), comprising three pivotal genes (*CD24*, *SPINT1*, and *QPRT*), was established through stepwise Cox regression analysis (**Figures 1I, J**).

### Validation of the TRPS in breast cancer

**Figure 2:**
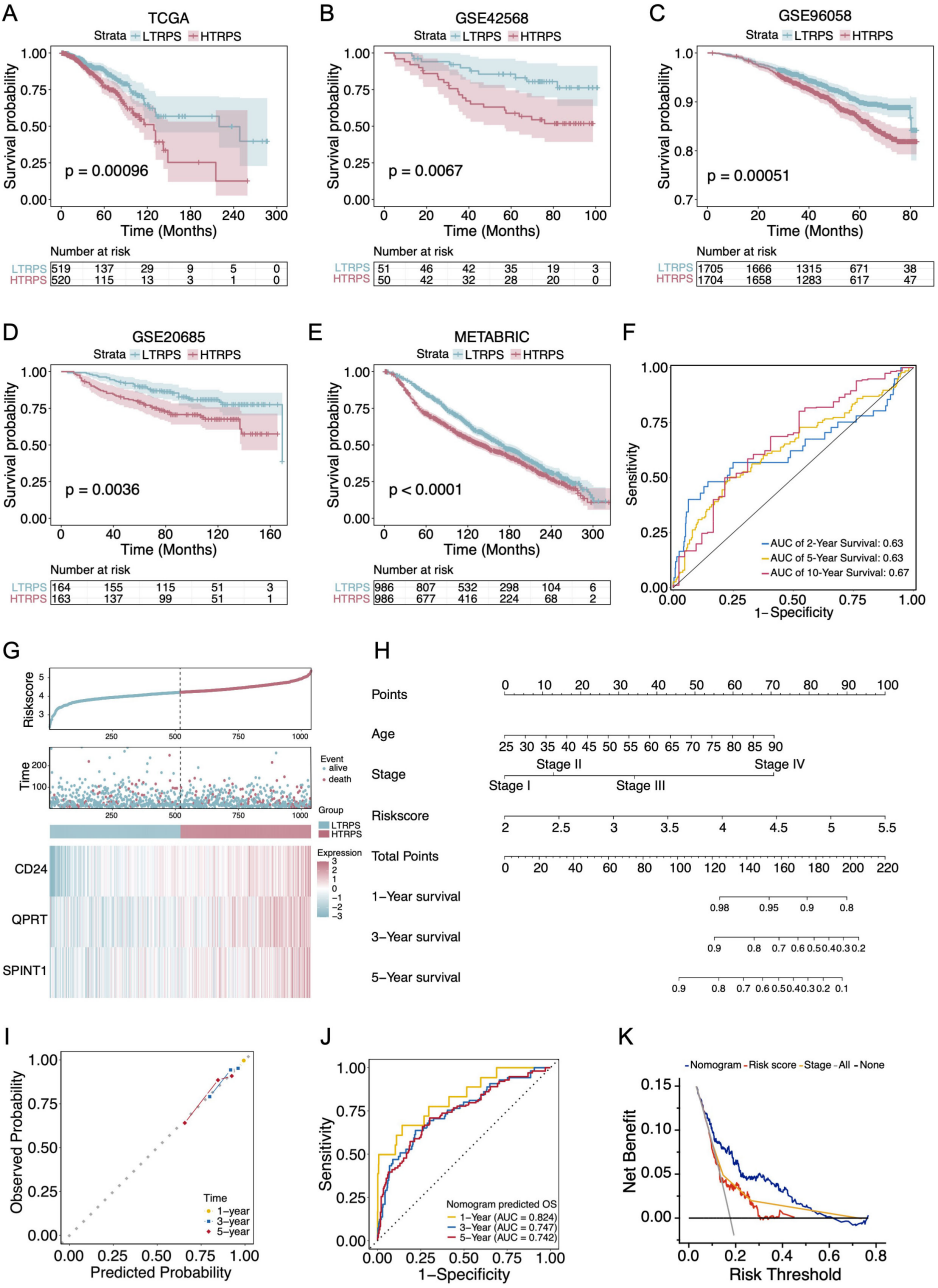
Validation and clinical utility of the TRPS model for prognosis prediction. **(A–E)** Kaplan-Meier survival analysis for OS between LTRPS and HTRPS groups in the TCGA-BC cohort **(A)**, and four independent external validation datasets: GSE42568 **(B)**, GSE96058 **(C)**, GSE20685 **(D)**, and METABRIC **(E)**. **(F)** Time-dependent ROC curves evaluating the predictive accuracy of the TRPS for 2-, 5-, and 10-year OS. **(G)** Distribution of risk scores (top), survival status of individual patients (middle), and expression patterns of signature genes (bottom). **(H)** Construction of a clinical nomogram integrating the TRPS risk scores with clinicopathological factors to predict 1-, 3-, and 5-year survival probabilities. **(I)** Calibration plots demonstrating the consistency between nomogram-predicted survival probabilities and actual observed outcomes at 1-, 3-, and 5-year intervals. **(J)** ROC curves evaluating the predictive accuracy of the nomogram for 1-, 3-, and 5-year survival probabilities. **(K)** Decision curve analysis assessing the net benefit of the nomogram across various thresholds.

To assess the robustness of the TRPS-based risk score in predicting prognosis, BC patients were stratified into HTRPS and LTRPS (high and low TRPS scores) groups based on the median risk score. Kaplan-Meier survival analysis demonstrated that the HTRPS group correlated with significantly worse OS compared with the LTRPS group in the TCGA-BC cohort (**Figure 2A**; *P* = 0.00096), which was further validated across four independent external datasets, including GSE42568, GSE96058, GSE20685, and METABRIC (**Figures 2B–E**; all *P* < 0.01). Moreover, the TRPS displayed favorable predictive accuracy, as evidenced by the time-dependent ROC analysis (**Figure 2F**). Analysis of the risk score distribution and survival status indicated that the HTRPS group correlated with increased mortality events and advanced stage, with three model genes highly expressed in this group (**Figure 2G**; **Supplementary Figure 2A**).

Subsequently, we performed univariate and multivariate Cox regression analyses to evaluate the prognostic significance of the model. The results confirmed that the TRPS was a robust and independent risk factor for OS (Multivariate HR = 2.38, *P* < 0.001; **Supplementary Figures 2B, C**). To facilitate clinical application, we developed a prognostic nomogram by integrating the TRPS score with age and stage to predict 1-, 3-, and 5-year OS probabilities (**Figure 2H**). Calibration plots showed a high degree of concordance between the nomogram-predicted and actual observed OS probabilities (**Figure 2I**). The nomogram exhibited robust predictive accuracy, with 1-, 3-, and 5-year AUCs reaching 0.824, 0.747, and 0.742, respectively (**Figure 2J**). Moreover, the DCA plot indicated that the nomogram yielded superior net benefit than individual clinical features alone (**Figure 2K**). Collectively, these results demonstrate that the TRPS model provides a robust framework for predicting the prognosis of BC.

### Characterization of the immunological landscape and drug sensitivity patterns associated with the TRPS

**Figure 3:**
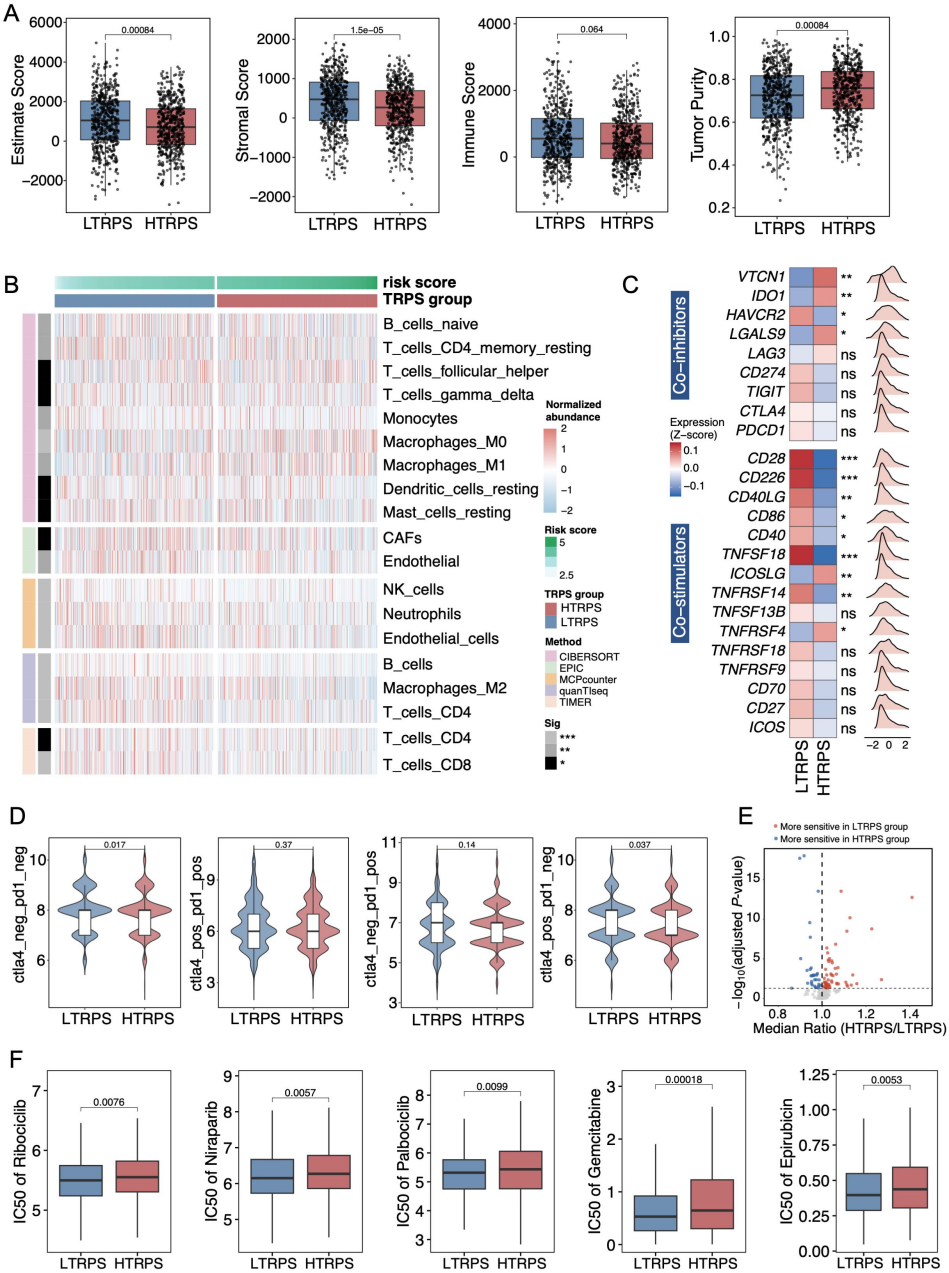
Immunological landscape and drug sensitivity analysis of TRPS-stratified risk groups. **(A)** Boxplots illustrating the comparison of Immune Score, ESTIMATE Score, Stromal Score, and Tumor Purity between LTRPS and HTRPS groups. **(B)** Heatmap showing the differential infiltration levels of immune cell populations evaluated by multiple deconvolution algorithms, including CIBERSORT, EPIC, MCPcounter, quanTIseq, and TIMER. **(C)** Expression profiles of key immune regulatory molecules between the LTRPS and HTRPS groups. **(D)** Violin plots comparing the IPS (immunophenoscore) values between the LTRPS and HTRPS groups. **(E)** Volcano plot visualizing the differential estimated drug sensitivity between LTRPS and HTRPS groups. The x-axis represents the median IC_50_ ratio (High/Low), and the y-axis represents -log_10_(*P*_adj_). Drugs with a ratio > 1 (red) and < 1 (blue) indicate resistance and sensitivity in the HTRPS group, respectively. **(F)** Boxplots comparing the IC_50_ values for five clinically relevant antitumor agents between the LTRPS and HTRPS groups. Wilcoxon rank-sum test was utilized. ^*^p < 0.05, ^**^p < 0.01, and ^***^p < 0.001, ns stands for not significant.

Next, we sought to explore the immunological features underlying the TRPS. The HTRPS group exhibited significantly lower Stromal (*P* = 1.5e-05) and ESTIMATE (*P* = 0.00084) scores, along with a marked increase in tumor purity score (*P* = 0.00084), reflecting a diminished infiltration of immune and stromal cells (**Figure 3A**). Specifically, deconvolution analysis of the tumor microenvironment (TME) revealed that T and B cells were significantly diminished in HTRPS patients (**Figure 3B**). Additionally, analysis of the expression of immune regulatory molecules showed that the LTRPS group displayed elevated expression of co-stimulatory molecules, while the HTRPS group exhibited significantly increased levels of co-inhibitory genes, such as *VTCN1* and *IDO1* (**Figure 3C**). Furthermore, IPS (immunophenoscore) analysis demonstrated higher values in the LTRPS group compared to the HTRPS counterpart, suggesting that LTRPS patients might be potentially more responsive to immunotherapy (**Figure 3D**). Lastly, we explored the therapeutic implications of the TRPS using the “oncoPredict” package. The volcano plot identified extensive differences in predicted IC_50_ values between the TRPS subgroups (**Figure 3E**). Notably, the HTRPS group exhibited reduced sensitivity (higher IC_50_ values) to some mainstay clinical agents, including Ribociclib, Niraparib, Palbociclib, Gemcitabine, and Epirubicin (**Figure 3F**). In summary, these data suggest the potential of TRPS as a predictive biomarker for both immune infiltration and therapeutic stratification.

### Genomic alterations and functional enrichment analysis associated with TRPS

**Figure 4:**
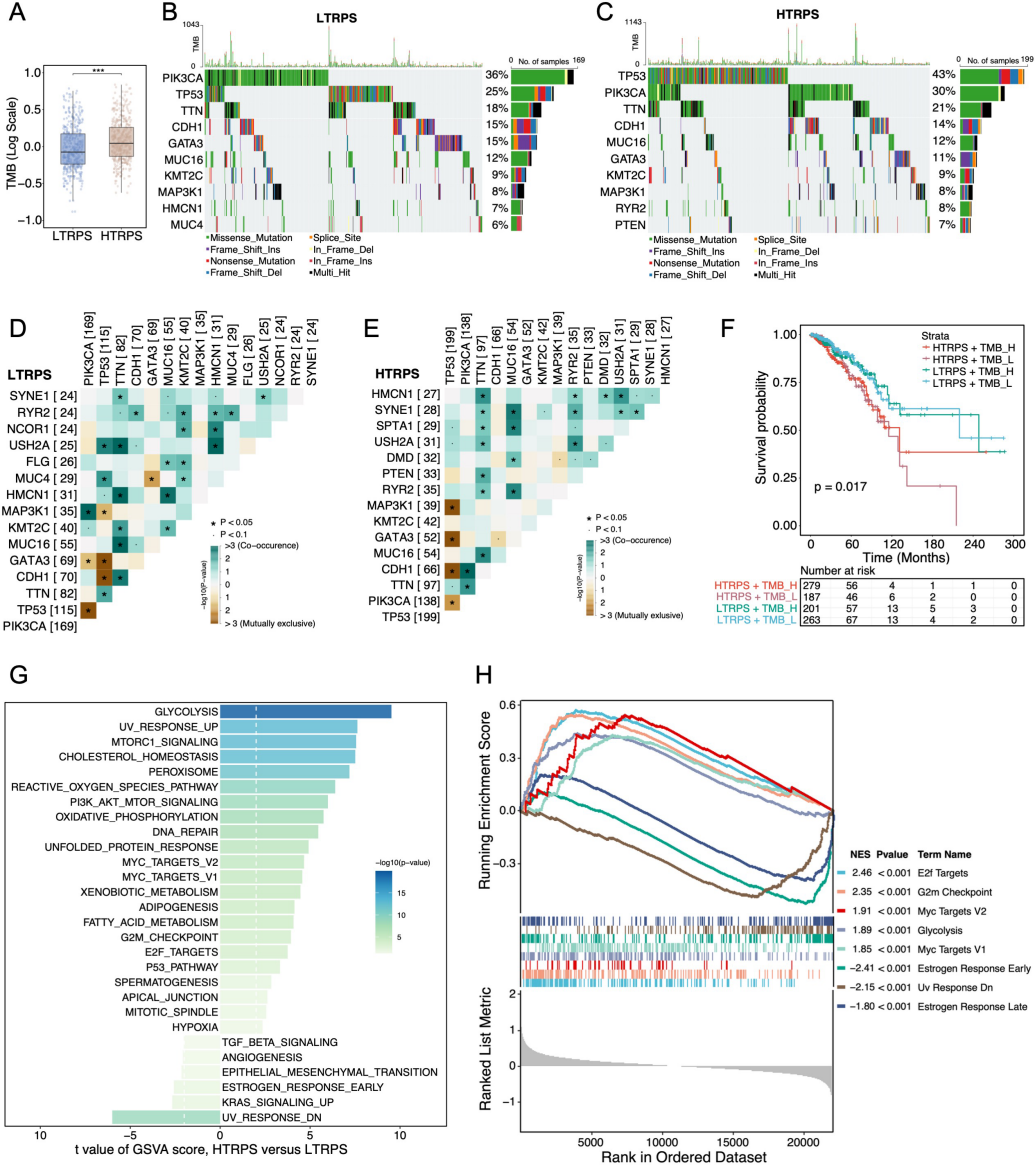
Genomic mutation landscapes and functional enrichment patterns associated with the TRPS. **(A)** Boxplot comparing the TMB levels between the LTRPS and HTRPS groups. The center line in each box represents the median; the box limits indicate the 25th and 75th percentiles. ^***^p < 0.001. **(B, C)** Oncoplots depicting the somatic mutation landscapes of the top 10 most frequently mutated genes in the LTRPS and HTRPS groups. **(D, E)** Heatmaps visualizing the mutual exclusivity and co-occurrence patterns among the top 15 mutated genes in the LTRPS and HTRPS groups. **(F)** Kaplan-Meier survival analysis for BC patients stratified by the combined status of TRPS and TMB status. **(G, H)** The differential functional enrichment patterns between the LTRPS and HTRPS groups were compared through GSVA **(G)** and GSEA **(H)** analyses. Wilcoxon rank-sum test **(A)**, pair-wise Fisher’s exact test **(D, E)**, and log-rank test **(F)** were utilized.

To further investigate the genomic features associated with the TRPS, we initially evaluated the tumor mutation burden (TMB). The HTRPS group exhibited a significantly higher TMB compared with the LTRPS group (**Figure 4A**). Analysis of the somatic mutation landscape revealed distinct mutational profiles between the two subgroups. Specifically, *TP53* was predominantly mutated in the HTRPS group, while *PIK3CA* alterations were more frequent in the LTRPS group (**Figures 4B, C**). Furthermore, somatic interaction analysis identified a mutually exclusive pattern between *PIK3CA* and *TP53* in both groups (**Figures 4D, E**), suggesting potentially alternative roles of these driver mutations in BC progression. Notably, stratified survival analysis combining TRPS and TMB indicated that patients in the HTRPS/low-TMB group experienced the worst outcomes (**Figure 4F**). Functionally, GSVA and GSEA demonstrated marked enrichment in several oncogenic and proliferation-related pathways, including E2F targets, G2M checkpoint, MYC targets, and glycolysis in the HTRPS group (**Figures 4G, H**). Conversely, the LTRPS group exhibited enrichment in estrogen response pathways. Collectively, these genomic and functional findings offer insights into the potential molecular underpinnings of the TRPS.

### Single-cell analysis of the TRPS

**Figure 5:**
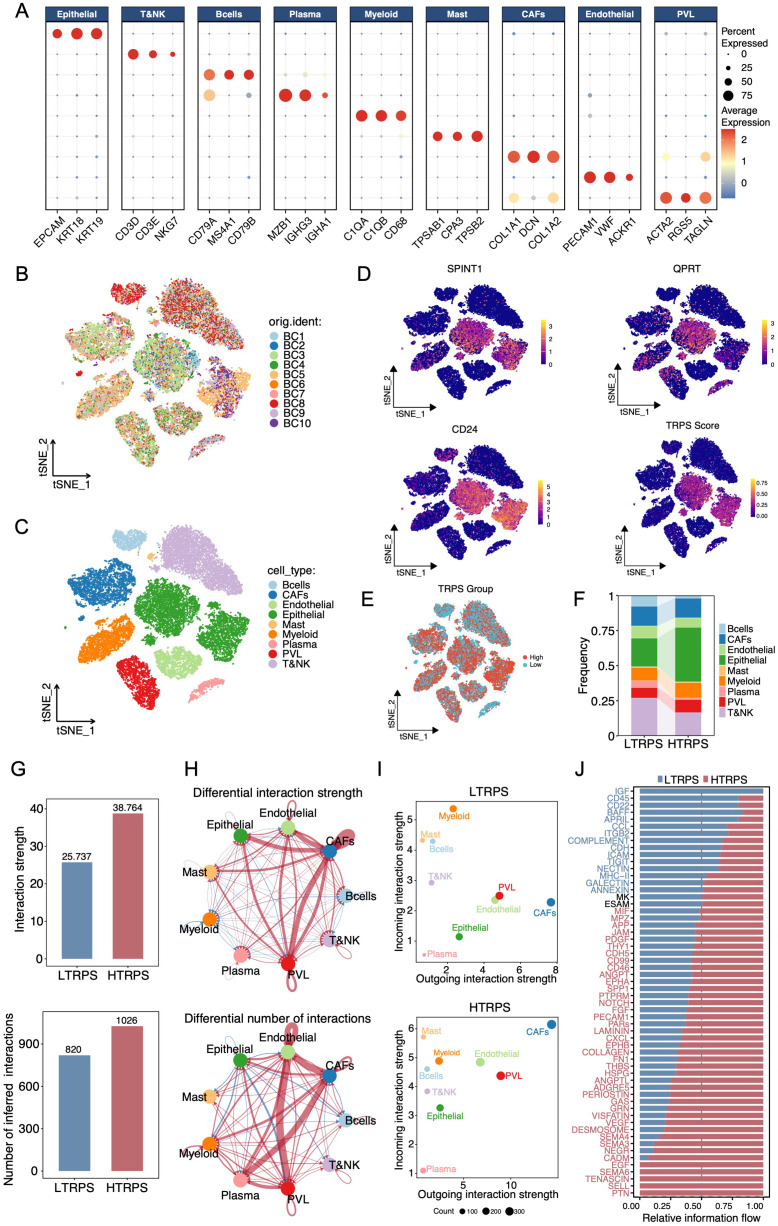
Single-cell landscape and intercellular communication network associated with the TRPS. **(A)** Dot plot illustrating the expression of canonical marker genes across nine major cell types. The dot size and color intensity represent the percentage of cells expressing the gene and the relative average expression level, respectively. **(B, C)** t-SNE visualization of major cell lineages, color-coded by patient identity **(B)** and cell lineage **(C)**. **(D)** Feature plots showing the expression distribution of the model genes and the overall TRPS score calculated via the AUCell algorithm. **(E)** t-SNE projection of cells into low- and high-TRPS groups. **(F)** Stacked barplot comparing the relative abundance of distinct cell populations between the LTRPS and HTRPS groups. **(G)** Barplots displaying the global differences in interaction strength (top) and number (bottom) of cell-cell communication between the two subgroups. **(H)** Circle plots visualizing differential interaction strength (top) and number (bottom) of cell-cell communication networks. Red lines indicate enhanced interactions in the HTRPS group. **(I)** Scatter plots depicting overall outgoing and incoming signaling patterns of various cell types in the LTRPS (top) and HTRPS (bottom) environments. **(J)** Comparison of the relative information flow of specific signaling pathways between the LTRPS and HTRPS groups.

To investigate the immune landscape associated with the TRPS framework at single-cell resolution, scRNA-seq was conducted on 10 primary breast tumor specimens. Following stringent quality control, a total of 29, 054 high-quality cells were obtained (**Supplementary Figure 3**). Subsequent unsupervised clustering and dimensionality reduction resolved nine major cell lineages, including epithelial cells, T/NK cells, B cells, PCs, myeloid cells, mast cells, cancer-associated fibroblasts (CAFs), endothelial cells, and perivascular-like (PVL) cells (**Figures 5A–C**). Notably, the TRPS score and individual model genes were predominantly expressed in epithelial cells (**Figures 5D, E**). Intriguingly, the HTRPS group displayed a notable decrease in the abundance of B cells, PCs, and T/NK cells (**Figure 5F**). Moreover, the HTRPS group exhibited significantly lower scores for the 12-chemokine TLS signature, 9-gene TLS signature, GC signature, and HEV signature compared to the LTRPS group (**Supplementary Figure 3F**), indicating that a high TRPS score is associated with a “TLS-deficient” TME. Meanwhile, CellChat analysis displayed enhanced global cellular interactions in the HTRPS group (**Figure 5G**). Specifically, analysis of differential cell-cell communication and centrality revealed a substantial increase in both the number and strength of interactions between epithelial cells and stromal components (including CAFs, endothelial cells, and PVL cells) in the HTRPS group, indicating an intensified tumor-stroma crosstalk within this subgroup (**Figures 5H, I**). Information flow analysis revealed that the HTRPS group displayed a prominent enrichment of pathways associated with malignant progression, stromal remodeling, and angiogenesis, including PTN, TENASCIN, VEGF, SEMA4, SPP1, COLLAGEN, and NOTCH, whereas the LTRPS group was predominantly enriched in immunostimulatory and chemotactic pathways, such as IGF, CD45, CD22, BAFF, APRIL, and CCL (**Figure 5J**). These signals are involved in B-cell survival and recruitment, lymphocyte activation, and immune surveillance. Collectively, these results indicate that TRPS is closely correlated with the immune environment and malignant progression in BC.

### QPRT correlates with poor prognosis and promotes BC progression

To evaluate the clinical significance of the genes within the TRPS model, we first characterized their stage-specific expression patterns and prognostic implications in BC patients. Among the three candidate genes, QPRT displayed the most pronounced stage-dependent upregulation and was most strongly correlated with a dismal prognosis, highlighting its pivotal role as a potential oncogenic driver in BC (**Supplementary Figures 4A, B**). Analysis of the TCGA BC datasets and the HPA database revealed that QPRT was significantly overexpressed in BC tissues compared to normal breast tissues (**Supplementary Figures 4C–E**). Moreover, Western blotting (WB) analysis confirmed that QPRT protein levels were remarkably elevated in both BC tissues and cell lines (**Supplementary Figures 4F, G**).

**Figure 6:**
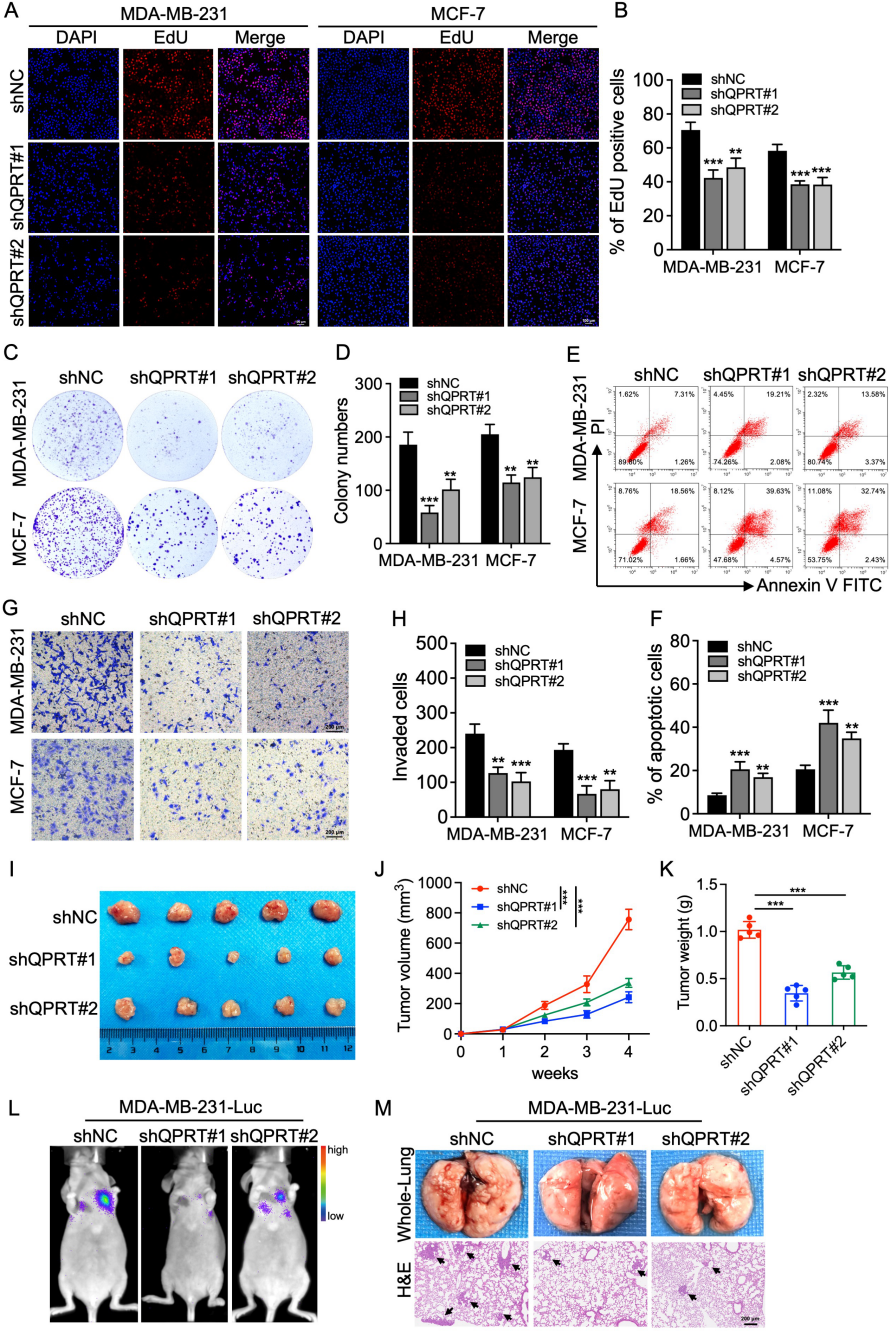
Knockdown of QPRT inhibits BC progression *in vitro* and *in vivo*. **(A, B)** Assessment of cellular proliferation through EdU incorporation in control and QPRT-knockdown BC cells. Scale bar: 100 μm. The barchart shows the percentage of EdU-positive cells. **(C, D)** Representative images and quantification of colony formation assays. **(E, F)** Representative images of Annexin V-FITC/PI apoptosis analysis by flow cytometry **(F, C)** and corresponding barchart quantifying the percentages of apoptotic cells. **(G, H)** Representative images of Transwell invasion assays were shown. Scale bar: 200 μm. The histograms indicate the average number of invaded cells per view. **(I–K)** *In vivo* tumor growth in an orthotopic xenograft model. Photographs of excised tumors **(I)**; tumor volume growth curves **(J)**; final tumor weights **(K)**. **(L)** Representative bioluminescence imaging (BLI) signals illustrating the lung metastatic burden in mice injected with QPRT-knockdown or control MDA-MB-231-Luc cells. **(M)** Photographs of dissected lungs (top) and representative images of H&E-stained lung tissue sections (bottom). Black arrows indicate metastatic nodules. Scale bar: 200 μm. Data are shown as mean ± SD. One-way ANOVA **(B, D, F, H, K)** and two-way ANOVA **(J)** were utilized. ^**^p < 0.01, ^***^p < 0.001.

To investigate the functional role of QPRT in BC progression, we subsequently established stable QPRT-knockdown and overexpression cell models via lentiviral transduction (**Supplementary Figures 4H, I**). Colony formation and EdU incorporation assays demonstrated that QPRT knockdown markedly inhibited the proliferative capacity of BC cells (**Figures 6A–D**), while its ectopic expression augmented BC cell proliferation (**Supplementary Figures 5A–D**). Flow cytometry (FC) analysis showed that loss of QPRT triggered an increase in cellular apoptosis (**Figures 6E, F**). Additionally, Transwell assay demonstrated that QPRT deficiency suppressed the invasive capability of BC cells (**Figures 6G, H**), whereas overexpression of QPRT facilitated BC cell invasion (**Supplementary Figures 5E, F**).

To validate these *in vitro* findings, orthotopic xenograft models were employed. The results showed that QPRT knockdown resulted in a significant reduction in both tumor volume and weight (**Figures 6I–K**), whereas overexpression of QPRT accelerated tumor growth (**Supplementary Figures 5G–I**). Furthermore, BC cells were injected into the tail vein to establish a metastasis model. Notably, loss of QPRT markedly reduced the lung metastatic burden (**Figures 6L, M**), while QPRT overexpression enhanced pulmonary metastasis (**Supplementary Figures 5J, K**). Altogether, these data collectively demonstrate that QPRT is highly expressed in BC patients and facilitates BC growth and metastasis.

### QPRT activates the Wnt/β-catenin signaling pathway and upregulates PD-L1 expression

**Figure 7:**
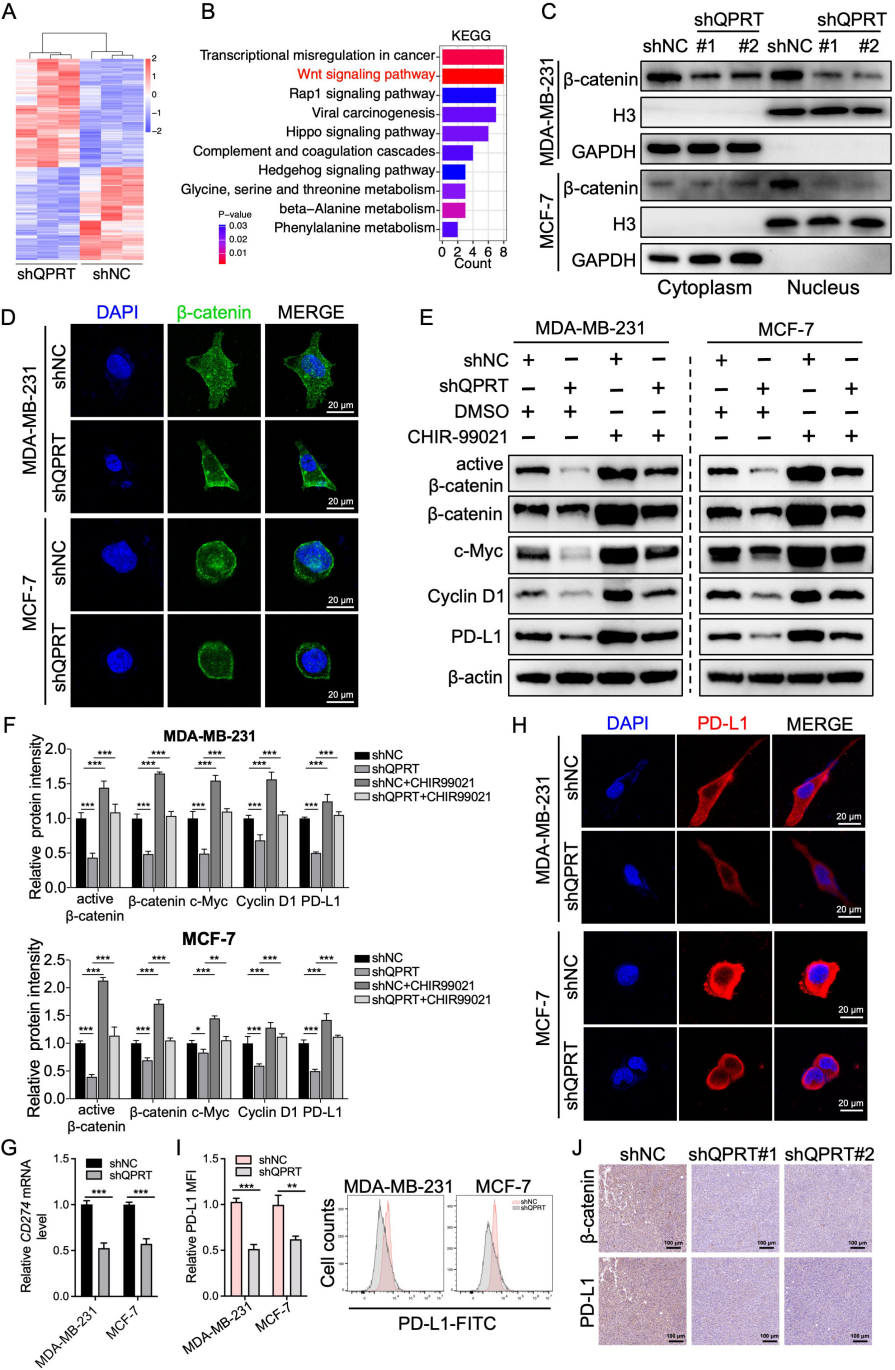
QPRT activates Wnt/β-catenin signaling and upregulates PD-L1 expression. **(A)** Heatmap illustrating DEGs identified by RNA-seq analysis between control and QPRT-knockdown BC cells. **(B)** KEGG pathway enrichment analysis of the DEGs showing significant involvement of the Wnt signaling pathway. **(C)** Western blot analysis of nuclear and cytoplasmic fractions of β-catenin in control and QPRT-knockdown BC cells. **(D)** IF staining of β-catenin in BC cells following QPRT silencing. Scale bars: 20 μm. **(E)** Western blot analysis of the levels of β-catenin pathway-associated proteins in control and QPRT-knockdown BC cells, with or without concurrent treatment with the Wnt agonist, CHIR-99021 (10 μM). **(F)** Quantification of protein levels in **(E, G).** qRT-PCR detecting *CD274* mRNA levels in control and QPRT-knockdown BC cells. **(H)** Representative IF staining of PD-L1 expression in control and QPRT-knockdown BC cells. Scale bars: 20 μm. **(I)** FC analysis of cell surface PD-L1 expression. **(J)** IHC staining of PD-L1 and β-catenin proteins in xenograft BC tumors. Scale bars: 100 μm. Data are shown as mean ± SD. One-way ANOVA **(F)** and Student t-test **(G, I)** were utilized. *p < 0.05, ^**^p < 0.01, ^***^p < 0.001.

To elucidate the underlying mechanisms by which QPRT contributes to BC progression, we performed RNA-seq on QPRT-knockdown and control cells (**Figure 7A**). KEGG pathway enrichment analysis identified the Wnt signaling pathway as a primary downstream target of QPRT (**Figure 7B**). Given that the nuclear translocation of β-catenin is a hallmark of Wnt activation (38), subcellular fractionation assays were conducted. The results showed that QPRT knockdown significantly impaired the nuclear accumulation of β-catenin (**Figure 7C**). This was further corroborated by immunofluorescence (IF) results, which showed a marked decrease in nuclear β-catenin upon QPRT silencing (**Figure 7D**). The Wnt/β-catenin signaling has been reported to upregulate PD-L1 expression in various cancers (39, 40). Consistently, QPRT deficiency led to a downregulation of Wnt pathway-associated proteins, including β-catenin, c-Myc, Cyclin D1, and PD-L1 (**Figures 7E, F**). Notably, treatment with the Wnt signaling agonist, CHIR-99021, effectively restored the protein levels of these targets in QPRT-knockdown BC cells (**Figures 7E, F**). Consistently, QPRT depletion diminished *CD274* (encoding PD-L1) mRNA levels (**Figure 7G**). Moreover, IF and FC analysis consistently confirmed that QPRT knockdown decreased PD-L1 protein levels in BC cells (**Figures 7H, I**). Notably, IHC analysis of xenograft tissues showed that β-catenin and PD-L1 were substantially downregulated following QPRT knockdown (**Figure 7J**). Correspondingly, CHIR-99021 treatment counteracted the inhibitory effects of QPRT knockdown on BC cell proliferation and invasion (**Supplementary Figure 6**). Taken together, these results demonstrate that QPRT orchestrates the Wnt/β-catenin/PD-L1 axis to promote BC progression.

### QPRT-mediated NAD^+^ accumulation stimulates the activation of Wnt/β-catenin signaling in a SIRT1-dependent manner

**Figure 8:**
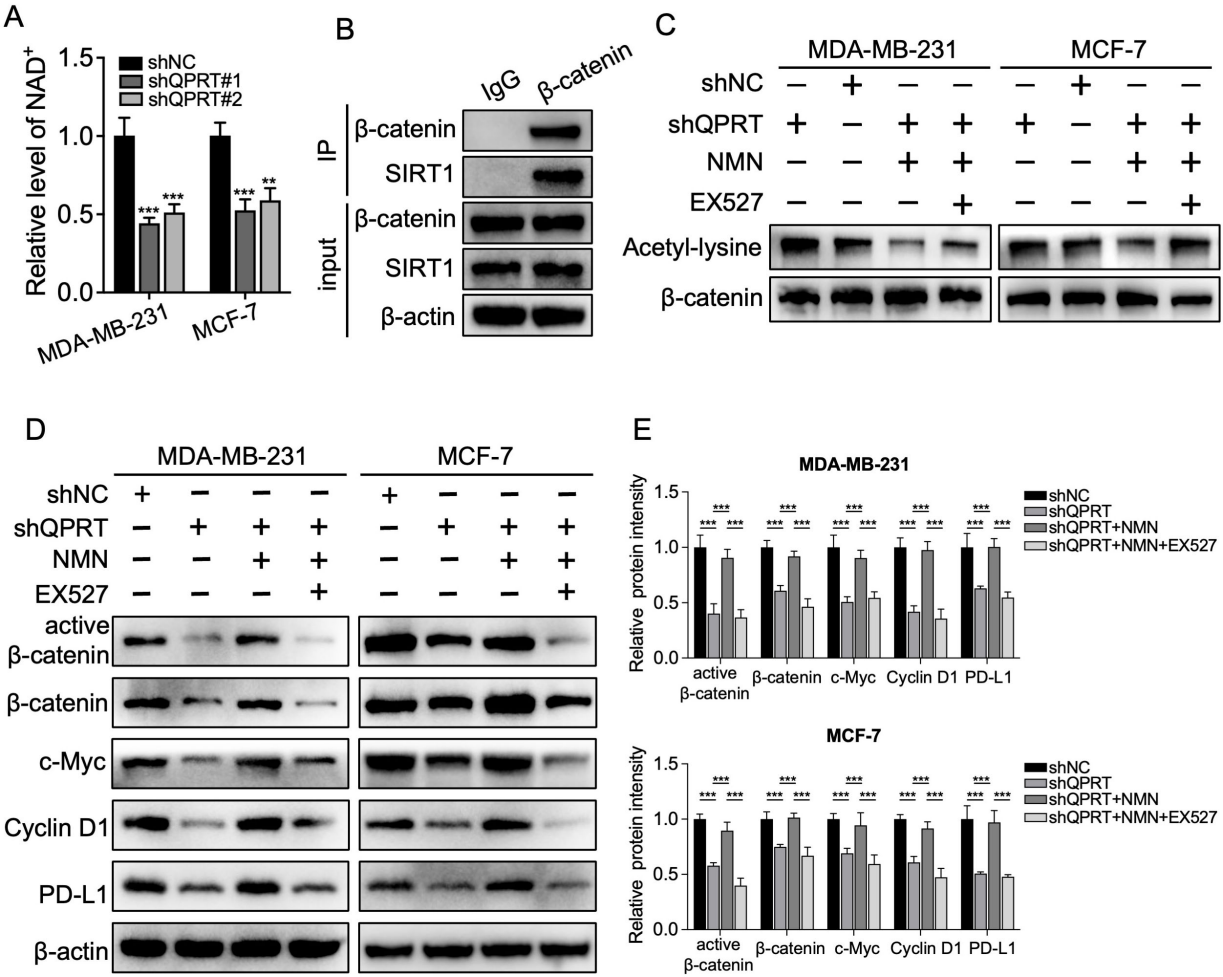
QPRT-mediated NAD^+^ accumulation induced activation of β-catenin pathway through SIRT1-mediated deacetylation. **(A)** Quantitative analysis of intracellular NAD^+^ content in control and QPRT-deficient BC cells. **(B)** Co-IP assay was conducted to detect the interaction between β-catenin and SIRT1 in MDA-MB-231 cells. **(C)** QPRT-knockdown BC cells were treated with the NAD^+^ precursor (NMN) (100 μM), with or without concurrent treatment with the SIRT1 specific inhibitor EX527 (20 μM), and the acetylation level of β-catenin was detected via immunoprecipitation assay. **(D)** Western blot analysis of the levels of β-catenin pathway-associated proteins. **(E)** Quantification of protein levels in **(D)** Data are shown as mean ± SD. One-way ANOVA was utilized. ^**^p < 0.01, ^***^p < 0.001.

Given that NAD^+^ serves as a critical substrate for the deacetylase SIRT1 (41), which in turn promotes the nuclear translocation and activity of β-catenin through deacetylation (42), we wondered whether QPRT regulates β-catenin signaling via the NAD^+^/SIRT1 axis. To this end, we first measured intracellular NAD^+^ levels. The results showed that NAD^+^ levels were significantly reduced in QPRT-deficient BC cells compared with control cells (**Figure 8A**). Moreover, Co-IP assay results confirmed the interaction between SIRT1 and β-catenin (**Figure 8B**), suggesting that SIRT1 may modulate the deacetylation of β-catenin in BC cells. To further determine whether QPRT-mediated NAD^+^ accumulation facilitates β-catenin deacetylation in a SIRT1-dependent manner, an immunoprecipitation (IP) assay was performed. As expected, the level of acetylated β-catenin (acβ-catenin) was increased in QPRT-knockdown BC cells, whereas exogenous supplementation of the NMN (a direct precursor of NAD^+^) resulted in decreased levels of acβ-catenin; notably, concurrent treatment with NMN and the SIRT1 selective inhibitor EX527 abolished the inhibitory effect of NMN on β-catenin acetylation (**Figure 8C**). Consistent with these findings, the levels of active and total β-catenin and its downstream targets were notably restored in NMN-treated QPRT-knockdown BC cells, and this restoration was reversed by EX527-induced inhibition of SIRT1 (**Figures 8D, E**). Taken together, these findings demonstrate that the QPRT/NAD^+^/SIRT1 axis promotes β-catenin deacetylation, thereby activating the Wnt/β-catenin pathway in BC.

### QPRT deficiency sensitizes BC to anti-PD-1 immunotherapy

**Figure 9:**
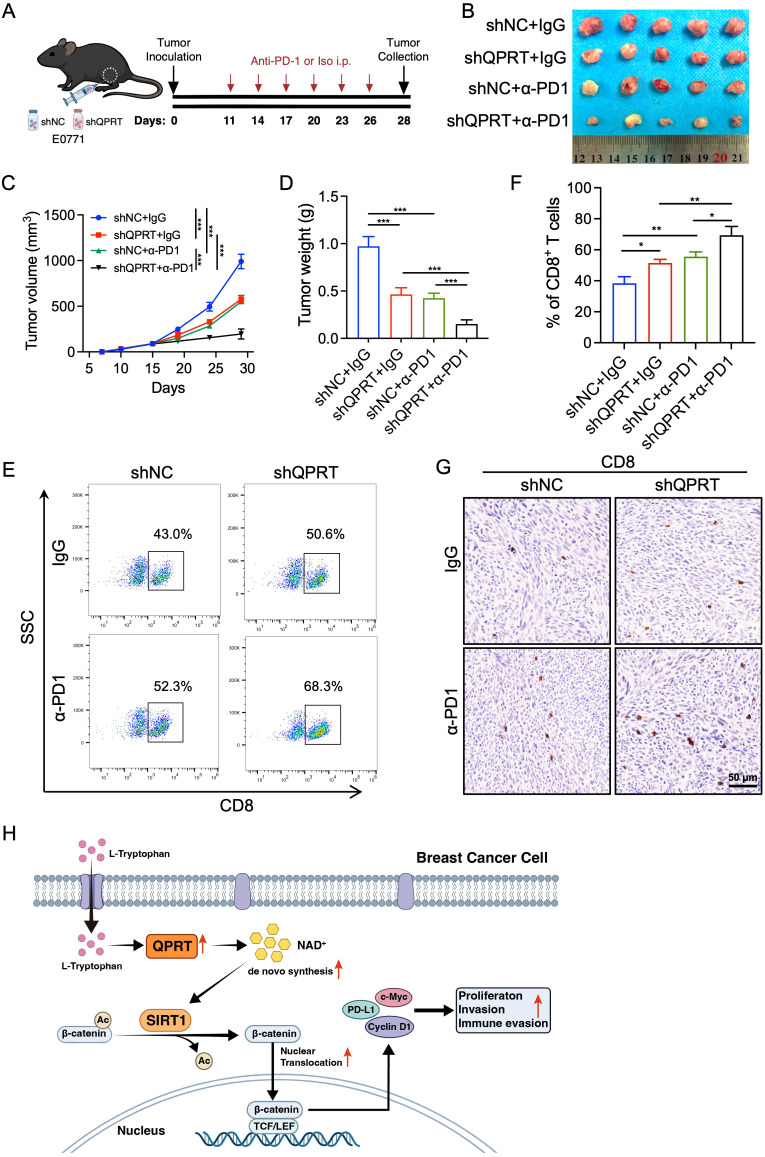
QPRT deficiency enhances CD8^+^ T cell infiltration and anti-PD-1 immunotherapy efficacy. **(A)** Schematic illustration of the immunotherapy regimen. After orthotopic inoculation of control or QPRT-knockdown E0771 cells, mice were intraperitoneally (i.p.) administered anti-PD-1 or IgG isotype control antibodies every three days. **(B)** Photographs of excised tumor. **(C)** Tumor volume growth curves. **(D)** Tumor weights at the end-point. **(E, F)** FC analysis of the percentages of tumor-infiltrating CD8^+^ T cells. **(G)** Representative IHC images of CD8^+^ T cells in BC tissue sections. Scale bar: 50 μm. **(H)** Schematic model of the QPRT/NAD^+^/SIRT1/β-catenin/PD-L1 axis facilitating the progression of BC cells. Data are shown as mean ± SD. One-way ANOVA **(D, F)** and two-way ANOVA **(C)** were utilized. ^*^p < 0.05, ^**^p < 0.01, ^***^p < 0.001.

Since PD-L1 upregulation in cancer cells mediates immune evasion and confers resistance to immune checkpoint inhibitors (43), we next investigated whether QPRT depletion could enhance the therapeutic efficacy of anti-PD-1 antibodies in a syngeneic orthotopic BC model (**Figure 9A**). Remarkably, combination therapy of QPRT knockdown with anti-PD-1-based immunotherapy yielded superior tumor control efficiency compared to either monotherapy, as evidenced by significantly reduced tumor volumes and weights (**Figures 9B–D**). Mechanistically, FC analysis revealed that the combination treatment group harbored the highest percentage of CD8^+^ T cells (**Figures 9E, F**). Consistently, IHC staining showed a marked increase in the density of CD8^+^ T cells in the combination treatment group (**Figure 9G**). Altogether, these findings suggest that QPRT deficiency might synergize with anti-PD-1 treatment in BC.

## Discussion

Therapy resistance remains an unresolved challenge in the clinical management of breast cancer (BC) (44). Importantly, TLSs in cancer are emerging as powerful predictors of favorable clinical outcomes upon immunotherapy (45, 46). In this study, we developed a novel TLS-related prognostic signature using machine learning methods through integrative analysis of single-cell and bulk transcriptomic data of BC. The established TRPS model effectively stratified BC patients into distinct prognostic subgroups and demonstrated clinical relevance. Compared with the LTRPS group, the HTRPS group was characterized by diminished immune infiltration, elevated TMB and enrichment of tumor-promoting signaling. Moreover, the HTRPS group exhibited a marked reduction in PCs, B cells, and T cells. Notably, we revealed that key model gene, QPRT, facilitated BC progression and immune evasion via the NAD^+^/SIRT1/β-catenin/PD-L1 axis (**Figure 9H**).

TLSs serve as pivotal immune niches for the interaction between immune cells, which fosters sustained anti-tumor immunity (47). For instance, the synergy between B cells and tissue-resident memory CD8^+^ T cells within TLSs potentiates PD-1 blockade efficacy in gastric cancer. Consistently, analysis of in-house scRNA-seq data revealed that T/NK cells, PCs and B cells were markedly enriched in the LTRPS group. Interestingly, while the HTRPS group exhibited a reduction in immune cell infiltration, CellChat analysis revealed a higher global interaction strength in these patients, indicating a rewiring of the TME communication landscape. In the HTRPS group, the heightened interaction strength is primarily driven by intensified crosstalk between epithelial cells and the stromal compartment (e.g., CAFs, PVL, and endothelial cells), rather than immune-related signaling. This is supported by the enrichment of pro-tumorigenic pathways such as PTN, SPP1, and COLLAGEN in the HTRPS group, which are known to be associated with extracellular matrix remodeling and angiogenesis. For instance, the upregulation of TENASCIN and COLLAGEN signaling may reflect a dense fibrotic stroma that physically impedes the trafficking and positioning of T and B cells into the tumor core. Similarly, the enrichment of VEGF, PTN, and NOTCH signaling likely signifies active angiogenesis. Conversely, the LTRPS group exhibited higher information flow in immunostimulatory pathways such as BAFF, APRIL, and CCL. The enrichment of these B-cell survival factors and chemotactic cues suggests a microenvironment conducive to lymphocyte recruitment and survival. Furthermore, differential expression analysis revealed that immune co-inhibitory molecules were predominantly upregulated in the HTRPS group, whereas co-stimulatory molecules were largely downregulated, suggesting a profound immunosuppressive state in HTRPS patients. In sum, our findings demonstrate that a high TRPS score is linked to an immunosuppressive TME in BC patients.

A growing body of evidence suggests that tumor cells reshape their TME through multiple mechanisms. For example, SETD2 loss in pancreatic tumor cells fosters the differentiation of CAFs into a lipid-laden phenotype, thereby sustaining tumor growth via OXPHOS (48). A recent study reported that tumoral-SLC6A6-mediated taurine deficiency promotes immune evasion by inducing T cell exhaustion (49). Tumor-mediated tryptophan metabolism forms a restrictive TME and deviates TLS maturation in hepatocellular carcinoma (34). These findings highlight the critical role of tumor-intrinsic factors in TME remodeling and TLS status, yet their association with TLSs in BC has largely been overlooked. Herein, we shifted the focus onto the previously undefined tumor-intrinsic factors associated with TLSs, and developed a novel TLS-related prognostic model (TRPS) comprising three genes (CD24, SPINT1, and QPRT). that exhibited robust prognostic predictive performance across four independent external BC cohorts. The clinical utility of the model was further validated through construction of a prognostic nomogram integrating TRPS and clinical features, which provides a potential tool for survival prediction in BC patients. Concurrently, we observed that *TP53*, a biomarker of genomic instability and poor prognosis in BC (50), was predominantly mutated in the HTRPS group, further underscoring the model’s reliability. Notably, each TRPS model gene demonstrated a significant correlation with shortened OS in BC. Cluster of differentiation 24 (CD24), a small and heavily glycosylated protein anchored to the cell membrane via glycosyl-phosphatidylinositol (GPI), is primarily expressed by immune cells and is also frequently overexpressed in various human tumors (51). Increasing studies have suggested that CD24 functions as a critical regulator of cell proliferation, migration, and invasion in various cancers. Recently, CD24 on tumor cells has been shown to facilitate an immunosuppressive milieu by interacting with the inhibitory receptor Siglec-10 on tumor-associated macrophages, positioning CD24 as a novel immune checkpoint and a promising target for cancer immunotherapy (52). Serine peptidase inhibitor, Kunitz type 1 (SPINT1), also known as HAI-1, is a membrane-bound serine protease inhibitor that plays a pivotal role in maintaining epithelial integrity (53, 54). Recently, Wu et al. demonstrated that SPINT1 was significantly overexpressed in BC patients and was relatively higher in those with lymphatic dissemination; furthermore, functional enrichment analysis revealed that the common co-expressed genes of SPINT1/2 were primarily associated with cell attachment and migration (55). Therefore, the TRPS model and its constituent genes hold promise as biomarkers for BC patient stratification.

Among the three TRPS model genes, QPRT exhibited the most pronounced association with the clinical advancement of BC, underscoring its critical role in BC malignant progression. QPRT is a bottleneck enzyme in the NAD^+^ *de novo* biosynthetic pathway. NAD^+^ exerts profound influence over diverse signal transduction processes by functioning as an essential substrate for various cellular enzymes such as sirtuins and Poly(ADP-ribose) polymerases (PARPs) (56). For instance, NAD^+^ promotes the nuclear translocation of PD-L1 by inducing SIRT1-mediated deacetylation at the H3K27 and H3K36 sites, thereby conferring resistance to immunotherapy in cervical cancer (57). Herein, we revealed that QPRT-mediated intracellular accumulation of NAD^+^ promotes the deacetylation of β-catenin in a SIRT1-dependent manner, ultimately stimulating the activation of the canonical Wnt/β-catenin signaling pathway and upregulating PD-L1 expression in BC cells. Notably, Lv et al. illustrated that dysregulated NAD^+^ metabolism drives tumor immune evasion in a CD8^+^ T cell-dependent manner in multiple tumors (58). Extending these observations, our findings showed that knockdown of QPRT significantly enhanced intratumoral CD8^+^ T cell infiltration. This suggests that the QPRT-NAD^+^ axis may function as a metabolic checkpoint that restricts the recruitment and functions of lymphocytes. Collectively, our findings, integrated with established evidence, indicate that QPRT may be implicated in the regulation of TLSs by modulating the CD8^+^ T cell-driven inflammatory milieu in BC. Notably, several potent inhibitors of phosphoribosyltransferase (NAMPT), the rate-limiting enzyme of the salvage pathway of NAD^+^ synthesis, have been developed exhibited robust anti-tumor activity in preclinical cancer models (59). Nevertheless, despite good tolerance and low toxicity, NAMPT inhibitors have failed to achieve satisfactory therapeutic outcomes in clinical trials (60). This discrepancy suggests that alternative NAD^+^ biosynthetic pathways may also play critical roles in tumor progression. Indeed, it has been reported that QPRT-mediated NAD^+^ synthesis alleviates oxidative stress in glioma, causing resistance to radiotherapy and chemotherapy (61). In this study, we found that QPRT depletion enhances sensitivity of BC to anti-PD-1 immunotherapy, highlighting QPRT as a promising therapeutic target to overcome resistance to ICIs in BC.

Several limitations should be noted for the present study. Firstly, the prognostic relevance of the TRPS model was validated using retrospective cohorts. Prospective studies will be required for rigorous assessment and refinement of its clinical utility. Secondly, the impact of QPRT-mediated NAD^+^ metabolism warrants further validation in clinical BC specimens. Lastly, the direct regulatory role of QPRT on CD8^+^ T cell infiltration remains to be defined. We will delve deeper into the precise mechanisms by which QPRT modulates immune evasion in future studies.

## Conclusion

In summary, this study developed and validated a robust TRPS-based prognostic signature for BC based on single-cell and bulk transcriptomic data through machine learning algorithms. The established model demonstrated high accuracy in prognostic prediction across multiple cohorts. A high TRPS score is associated with an immunosuppressive TME, as characterized by diminished infiltration of B cells, NK/T cells and PCs. Particularly, our findings suggest the potential of QPRT as a promising therapeutic target for sensitizing BC to immunotherapy. Overall, the TRPS model provides a novel framework for improving risk stratification and personalized clinical management of BC patients.

## Data Availability

The RNA-seq data supporting the conclusions of this article were deposited in the NCBI Sequence Read Archive (SRA) database (Accession number: PRJNA1458329).
