# Supplementary figures and images for: Development and validation of a TLS-associated signature for prognosis prediction in breast cancer: new insights into QPRT
Source: Front Immunol. 2026 May 7;17:1834127. doi: 10.3389/fimmu.2026.1834127 (PMC13189804; doi:10.3389/fimmu.2026.1834127)

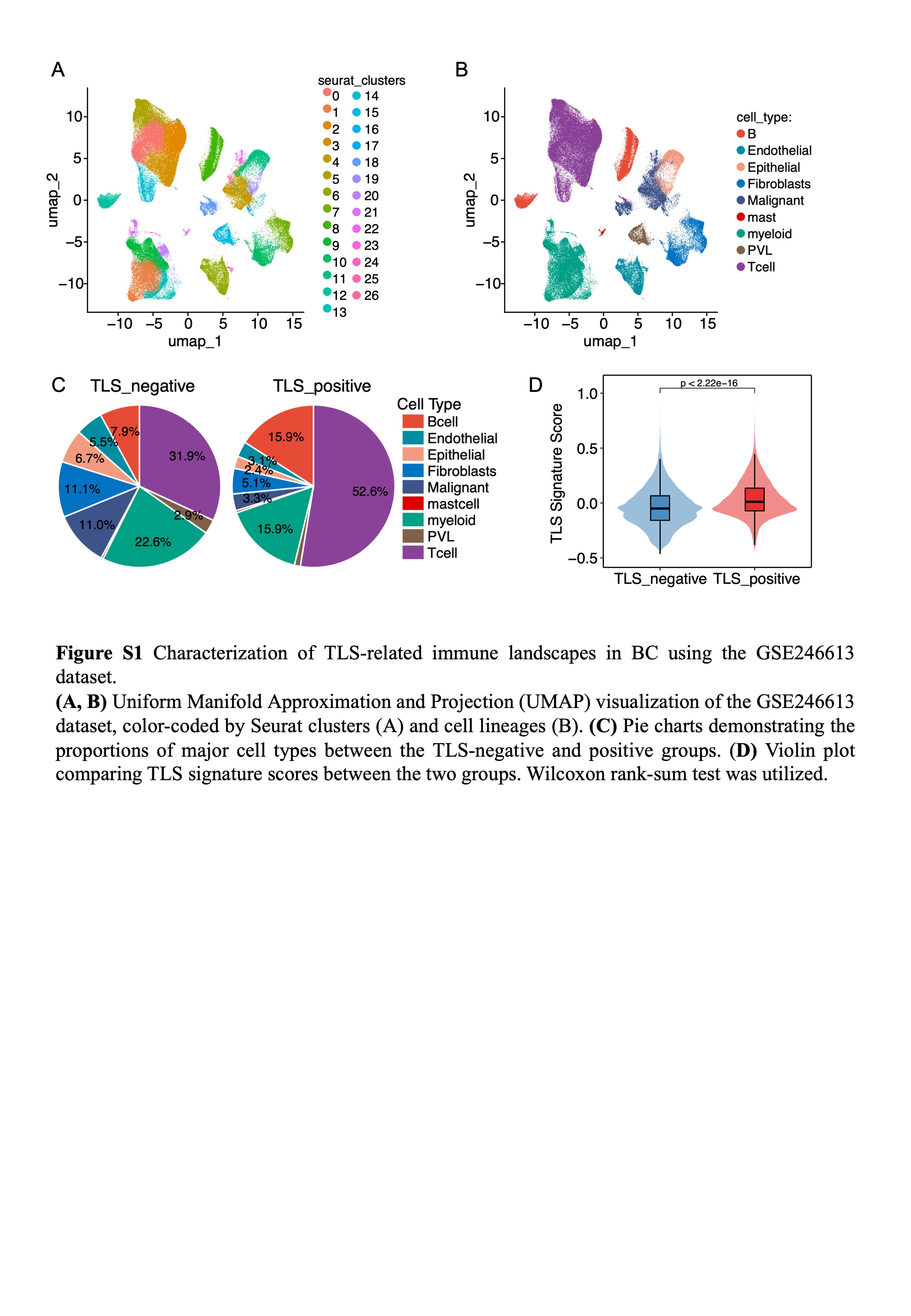

Supplement: Supplementary file 2 [file Image1.jpeg]

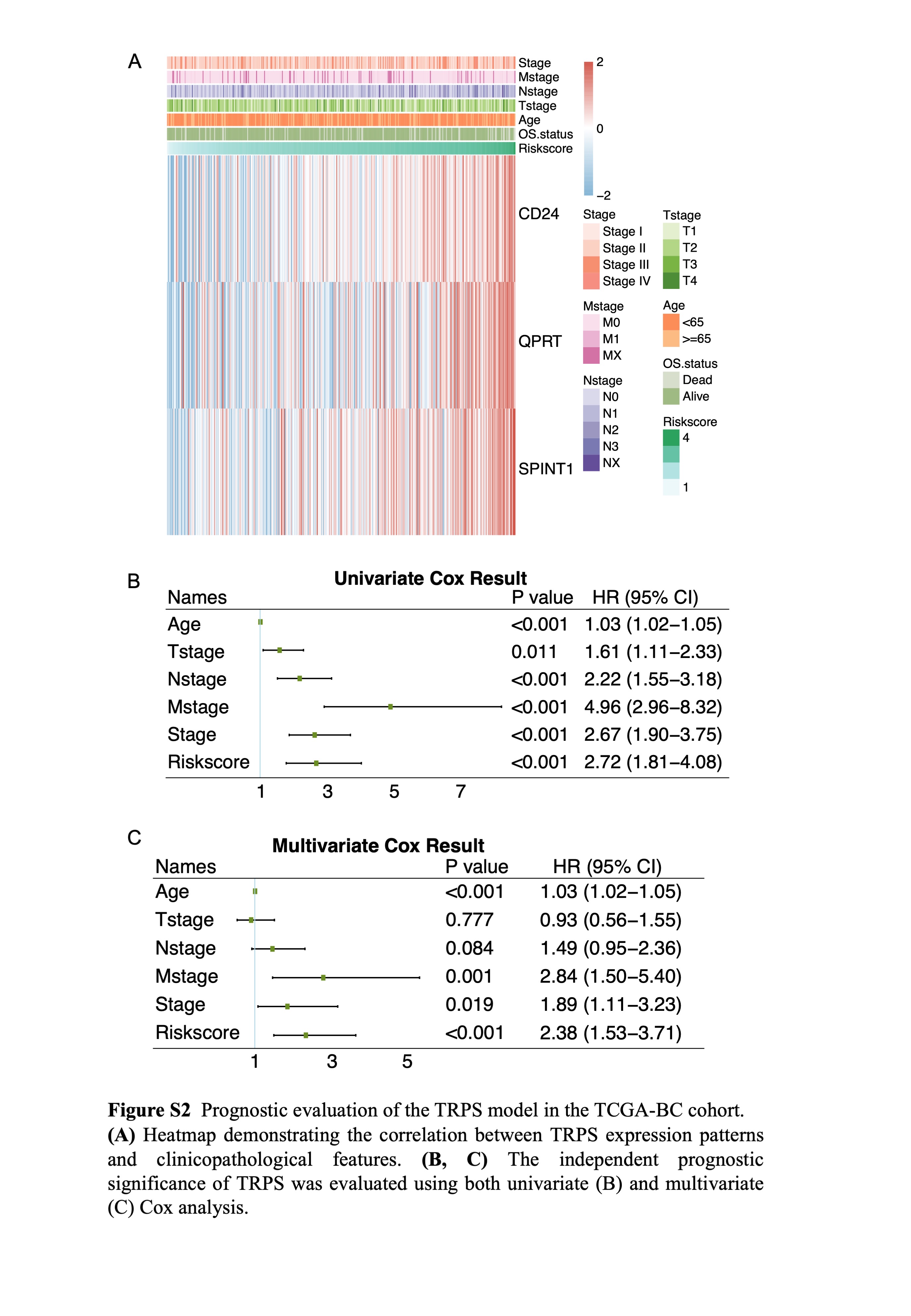

Supplement: Supplementary file 3 [file Image2.jpeg]

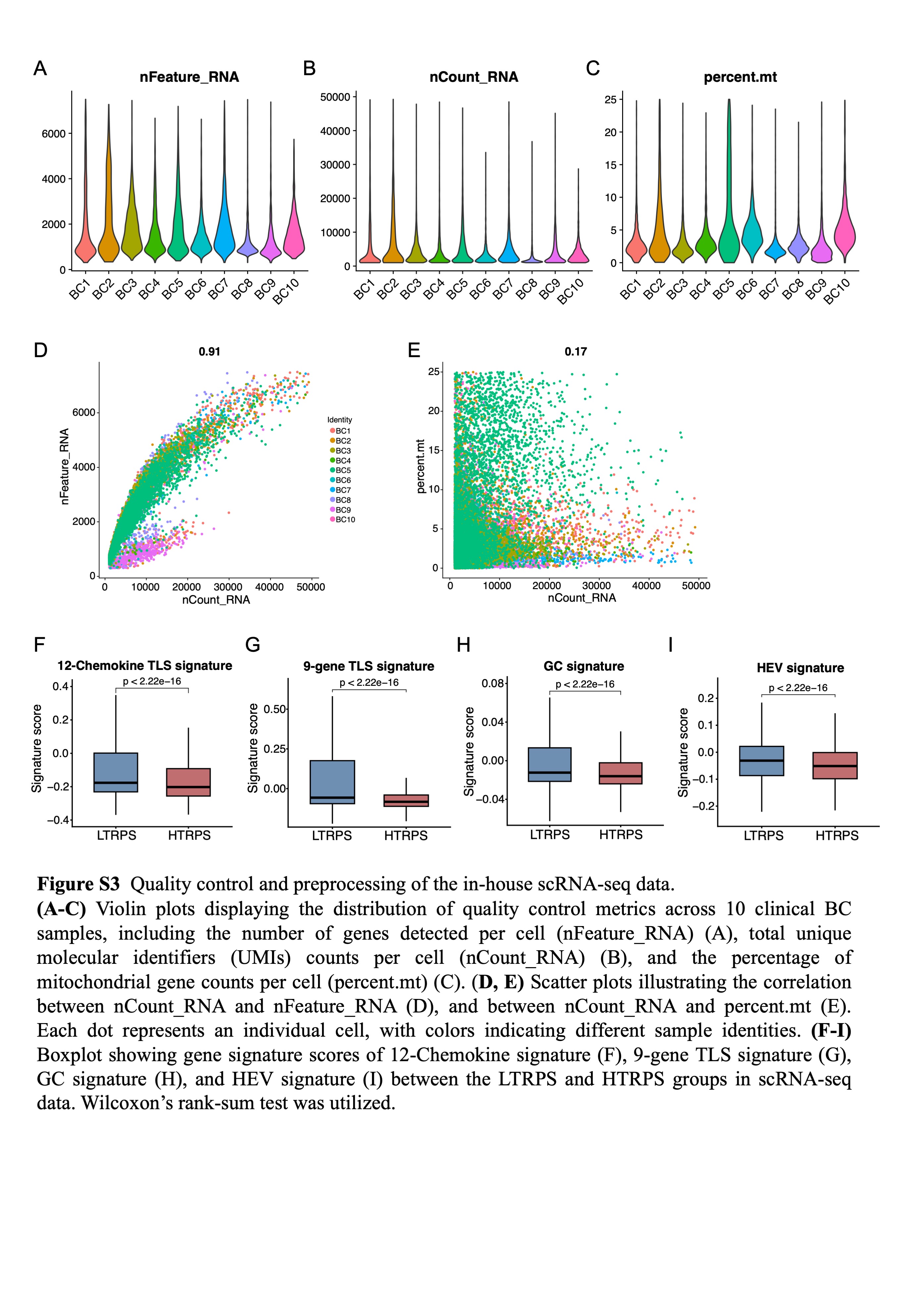

Supplement: Supplementary file 4 [file Image3.jpeg]

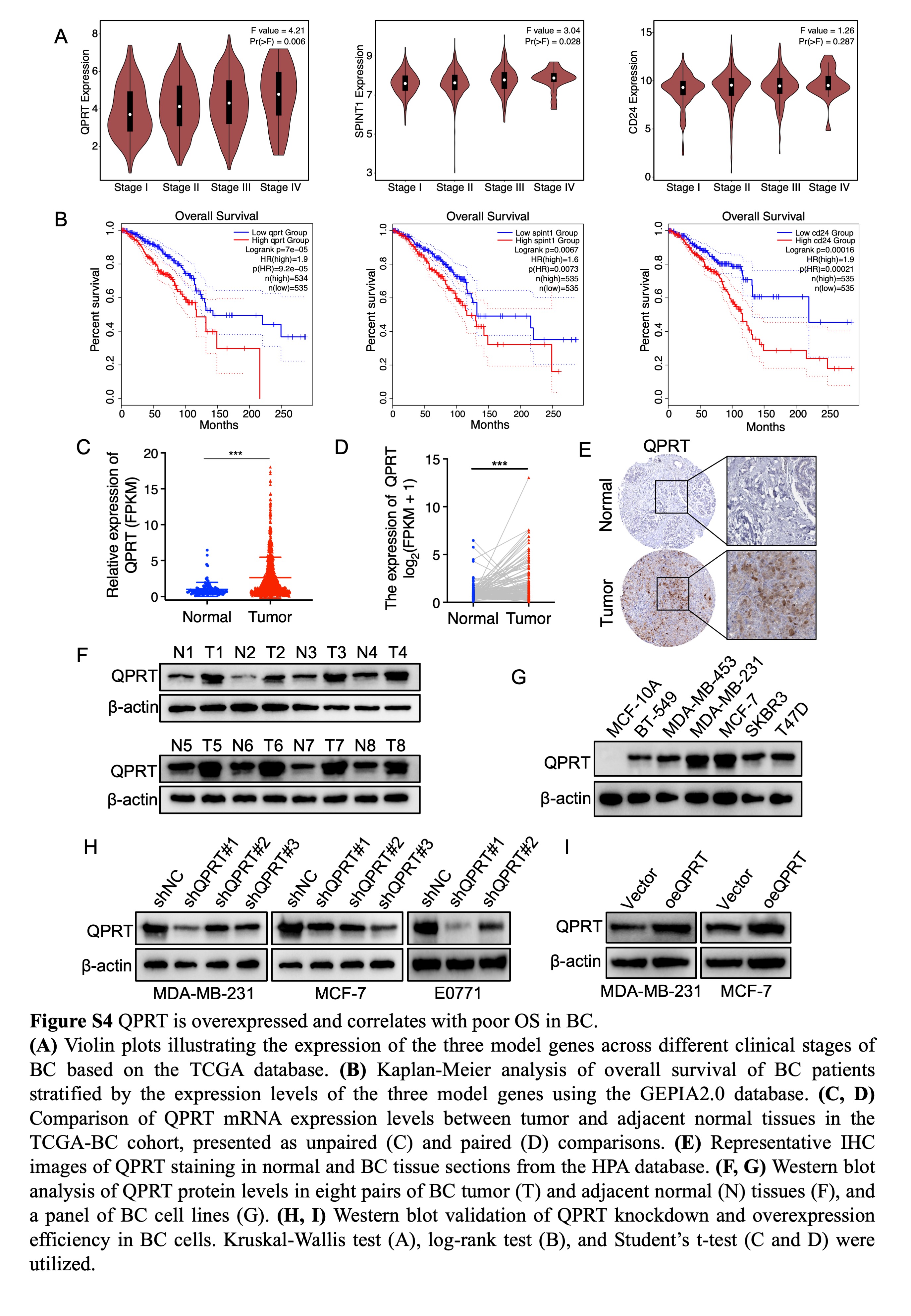

Supplement: Supplementary file 5 [file Image4.jpeg]

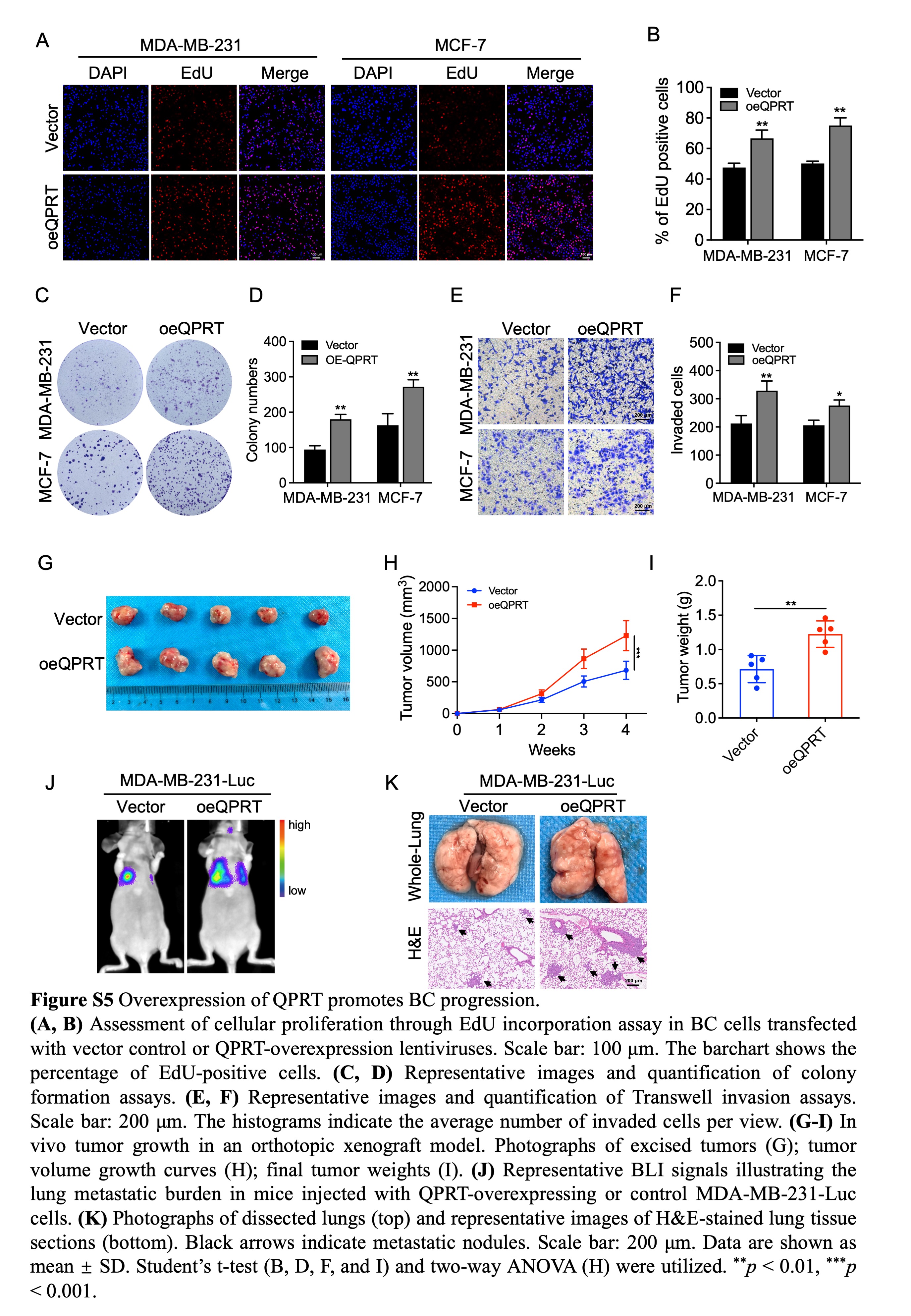

Supplement: Supplementary file 6 [file Image5.jpeg]

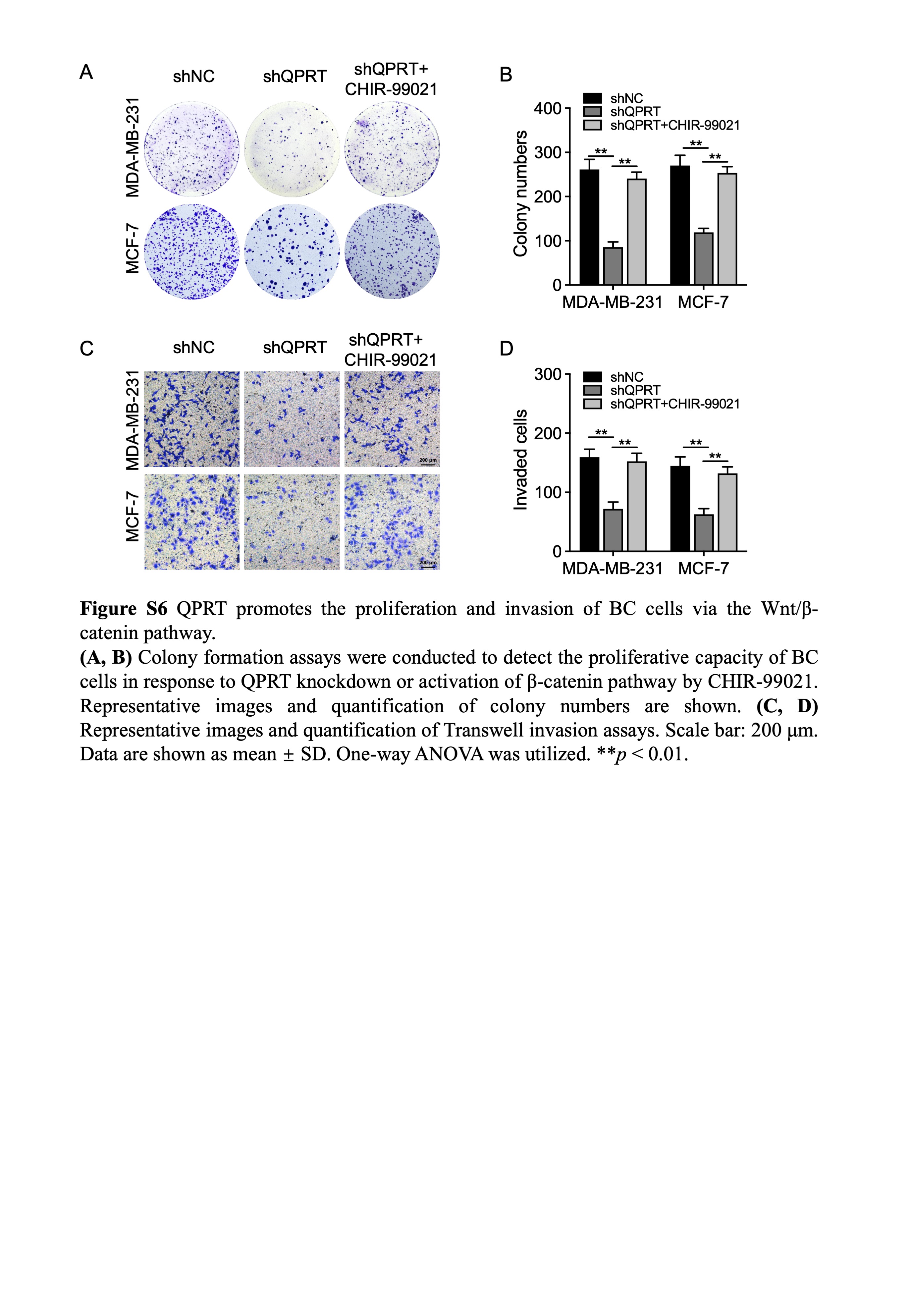

Supplement: Supplementary file 7 [file Image6.jpeg]
