# Supplementary material for: Development and validation of a TLS-associated signature for prognosis prediction in breast cancer: new insights into QPRT
Source: Front Immunol. 2026 May 7;17:1834127. doi: 10.3389/fimmu.2026.1834127 (PMC13189804; doi:10.3389/fimmu.2026.1834127)
Supplement: Supplementary file 8 [file Table1.docx]

**Table S2.** List of oligos used in this study

| **Name** | **Application** | **Sequence (5ʹ → 3ʹ)** | |
| --- | --- | --- | --- |
| shQPRT#1 (Homo) | Knockdown | Target site | GCTCATCTCAGTTTCCTAATC |
| shQPRT#2 (Homo) | Knockdown | Target site | ACCTTGTCCTGCTGGACAACT |
| shQPRT#3 (Homo) | Knockdown | Target site | GCACATTTGGCACTAGCTTGA |
| shQprt#1 (Mus) | Knockdown | Target site | CGAAGAAGATGCCACCAGAAT |
| shQprt#2 (Mus) | Knockdown | Target site | CGCCATCTTCACTCAACTCAA |
| PD-L1 | qRT-PCR | Forward | ATTTGCTGAACGCCCCATAC |
|  |  | Reverse | TCCAGATGACTTCGGCCTTG |
| β-actin | qRT-PCR | Forward | CCTGGCACCCAGCACAAT |
|  |  | Reverse | GGGCCGGACTCGTCATAC |
