# Supplementary material for: Development and validation of a TLS-associated signature for prognosis prediction in breast cancer: new insights into QPRT
Source: Front Immunol. 2026 May 7;17:1834127. doi: 10.3389/fimmu.2026.1834127 (PMC13189804; doi:10.3389/fimmu.2026.1834127)
Supplement: Supplementary file 9 [file Table2.docx]

**Table S3.** List of antibodies used in this study

| **Antibodies** | **Source** | **Cat.No** | | **Application** |
| --- | --- | --- | --- | --- |
| Purified Anti-Mouse CD16/32 | Elabscience | | E-AB-F0997A | FC |
| FITC Anti-Mouse CD45 | Elabscience | | E-AB-F1136C | FC |
| Alexa Fluor® 700 anti-mouse CD3 | BioLegend | | 100216 | FC |
| PerCP Anti-Mouse CD8a | Elabscience | | E-AB-F1104F | FC |
| FITC Anti-Human PD-L1 | Elabscience | | E-AB-F1133C | FC |
| QPRT | Proteintech | | 25174-1-AP | WB |
| β-Actin | Proteintech | | 66009-1-Ig | WB |
| Cyclin D1 | Proteintech | | 60186-1-Ig | WB |
| c-Myc | Proteintech | | 67447-1-Ig | WB |
| PD-L1 | Proteintech | | 66248-1-Ig | WB/IF/IHC |
| β-Catenin | CST | | 8480T | WB/IHC/IF |
| Non-phospho (Active) β-Catenin | CST | | 8814T | WB |
| HRP goat anti-rabbit IgG (H+L) | ABclonal | | AS014 | WB |
| CoraLite 594-conjugated Goat Anti-Mouse IgG (H+L) | Proteintech | | SA00013-3 | IF |
| CoraLite® Plus 488-Goat Anti-Rabbit Recombinant Secondary Antibody (H+L) | Proteintech | | RGAR002 | IF |
| CD8a | ABclonal | | A23081 | IHC |

**Abbreviations:** FC: flow cytometry; WB: western blot; IF: immunofluorescence; IHC: immunohistochemistry.
